# PKCδ-mediated SGLT1 upregulation confers the acquired resistance of NSCLC to EGFR TKIs

**DOI:** 10.1038/s41388-021-01889-0

**Published:** 2021-06-21

**Authors:** Chia-Hung Chen, Bo-Wei Wang, Yu-Chun Hsiao, Chun-Yi Wu, Fang-Ju Cheng, Te-Chun Hsia, Chih-Yi Chen, Yihua Wang, Zhang Weihua, Ruey-Hwang Chou, Chih-Hsin Tang, Yun-Ju Chen, Ya-Ling Wei, Jennifer L. Hsu, Chih-Yen Tu, Mien-Chie Hung, Wei-Chien Huang

**Affiliations:** 1 Division of Pulmonary and Critical Care Medicine, Department of Internal Medicine, China Medical University Hospital, Taichung, Taiwan; 2 School of Medicine, China Medical University, Taichung, Taiwan; 3 Department of Respiratory Therapy, China Medical University, Taichung, Taiwan; 4 Center for Molecular Medicine, Research Center for Cancer Biology, and Graduate Institute of Biomedical Sciences, China Medical University, Taichung, Taiwan; 5 Drug Development Center, China Medical University, Taichung, Taiwan; 6 The Ph.D. Program for Cancer Biology and Drug Discovery, China Medical University and Academia Sinica, Taichung, Taiwan; 7 Department of Biomedical Imaging and Radiological Sciences, National Yang-Ming University, Taipei, Taiwan; 8 Graduate Institute of Basic Medical Science, China Medical University, Taichung, Taiwan; 9 Department of Molecular and Cellular Oncology, The University of Texas MD Anderson Cancer Center, Houston, TX, USA; 10 Department of Internal Medicine, Hyperbaric Oxygen Therapy Center, China Medical University Hospital, Taichung, Taiwan; 11 Division of Thoracic Surgery, Department of Surgery, Institute of Medicine, Chung Shan Medical University, Taichung, Taiwan; 12 Biological Sciences, Faculty of Environmental and Life Sciences, University of Southampton, Southampton, UK; 13 Institute for Life Sciences, University of Southampton, Southampton, UK; 14 Department of Biology and Biochemistry, University of Houston, Houston, TX, USA; 15 Department of Medical Research, E-Da Hospital, Kaohsiung, Taiwan; 16 School of Medicine for International Students, I-Shou University, Kaohsiung, Taiwan; 17 Department of Pharmacy, E-Da Hospital, Kaohsiung, Taiwan; 18 Department of Medical Laboratory Science and Biotechnology, Asia University, Taichung, Taiwan

## Abstract

The tyrosine kinase inhibitors (TKIs) targeting epidermal growth factor receptor (EGFR) have been widely used for non-small cell lung cancer (NSCLC) patients, but the development of acquired resistance remains a therapeutic hurdle. The reduction of glucose uptake has been implicated in the anti-tumor activity of EGFR TKIs. In this study, the upregulation of the active sodium/glucose co-transporter 1 (SGLT1) was found to confer the development of acquired EGFR TKI resistance and was correlated with the poorer clinical outcome of the NSCLC patients who received EGFR TKI treatment. Blockade of SGLT1 overcame this resistance in vitro and in vivo by reducing glucose uptake in NSCLC cells. Mechanistically, SGLT1 protein was stabilized through the interaction with PKCδ-phosphorylated (Thr678) EGFR in the TKI-resistant cells. Our findings revealed that PKCδ/EGFR axis-dependent SGLT1 upregulation was a critical mechanism underlying the acquired resistance to EGFR TKIs. We suggest co-targeting PKCδ/SGLT1 as a potential strategy to improve the therapeutic efficacy of EGFR TKIs in NSCLC patients.

## Introduction

The epidermal growth factor receptor (EGFR), a membrane-bound tyrosine kinase receptor, has been found to be a critical oncogene in promoting the tumorigenesis, mitogenesis, and tumor progression of various cancer types, including non-small cell lung cancer (NSCLC) [1, 2]. Overexpression or somatic mutation of EGFR causes the aberrant activation of its tyrosine kinase and the dysregulation of its downstream signals that contribute to the tumor growth and progression and the poor prognosis of NSCLC patients [3]. EGFR is therefore a rational and feasible therapeutic target for this disease.

Small molecule tyrosine kinase inhibitors (TKIs) targeting the ATP-binding pocket of the EGFR kinase domain were developed and approved for NSCLCs [4]. Gefitinib (ZD1839, Iressa) and erlotinib (OSI-774, Tarceva) are two approved first-generation EGFR TKIs for NSCLC. However, these drugs fail to achieve maximum therapeutic efficacy, with response rates of typically 10–20% across a variety of malignancies [4]. Gefitinib and erlotinib particularly benefit Asian NSCLC patients who are female, never-smokers, and have adenocarcinoma histology. Importantly, the high prevalence of activating EGFR mutations, including the exon 19 (L746-A750) deletion and L858R point mutation, were found in these patients [5]. These activating mutations alter the protein structure of EGFR at its ATP-binding site and increase the binding affinity and vulnerability to TKIs [6]. However, a frequent substitution of threonine residue to methionine at codon 790 (T790M) of EGFR exon 20 has been found to reduce the binding affinity with gefitinib or erlotinib and thereby contributes to the development of acquired resistance [1]. The second-generation irreversible TKI, afatinib (BIBW2992, Gilotrif), was developed to target the activating EGFR mutant with less likelihood of the EGFR T790M point mutation [7]. The third-generation TKI, osimertinib (AZD9291, Tagrisso), was further designed to target the EGFR T790M mutation [8, 9]. The secondary EGFR mutations [10] and activations of alternative RTKs creating a bypass track [11, 12] have been proposed as reasons for the failed responses to EGFR TKIs, yet fail to fully account for the development of acquired resistance to these drugs in NSCLC patients.

Several studies have shown that, in addition to delivering the classic proliferation and survival signals, activated EGFR also facilitates glucose utility and metabolic pathways through the stabilization of glucose transporters and dysregulation of glycolytic enzymes hexokinases and pyruvate kinase M2 (PKM2) that promote tumor growth, epithelial-mesenchymal transition (EMT), cancer stemness, and even immune evasion [13–17]. Recently, tyrosine kinase activation of EGFR was shown to enhance the activity of 6-phosphofructo-2-kinase/fructose-2,6-bisphosphatase-3 (PFKFB3), an essential glycolytic activator for the synthesis and degradation of fructose-2,6-bisphosphate (F26BP) that contributes to the survival of NSCLC cells [18]. EGFR signaling also links glycolysis with serine synthesis to support nucleotide biosynthesis and redox homeostasis [19]. Mounting evidence has proved that EGFR impacts the rewiring of the glucose metabolic network to promote tumor progression in NSCLC [20].

Clinically, the decrease in glucose metabolism measured by the uptake of 2-[^18^F]-fluoro-2-deoxy-D-glucose ([^18^F]-FDG), the radiolabeled glucose analog, with positron emission tomography (PET) analysis appears within days of initiating EGFR TKI therapy in NSCLC patients [21]. [^18^F]-FDG uptake is significantly decreased by erlotinib treatment in sensitive, not insensitive, human NSCLC-xenograft mouse tumors [22, 23]. Changes in tumor glucose metabolism precede decreases in tumor size in response to EGFR TKIs [22, 24]. These findings suggest that reduction of EGFR-mediated glycolysis may be involved in the anti-tumor activity of EGFR TKIs, and thus monitoring [^18^F]-FDG-PET uptake has been used to predict therapeutic responses to EGFR TKIs in lung cancer patients [25–27]. Moreover, suppression of facilitative glucose transporter Glut was found to mediate the anti-cancer activity of TKIs in NSCLC cell lines bearing wild-type (wt) or mutant EGFR [28]. These studies suggested that the suppression of glucose uptake and metabolism may be essential to achieve the therapeutic response of EGFR TKIs in NSCLC patients. However, elevated lactate secretion and glycolysis were found in long-term TKI treatment of NSCLC cells and in prostate cancer cells [29–31], raising the possibility that glucose metabolic re-wiring may contribute to the development of acquired resistance of NSCLC to EGFR TKIs.

In this study, our data showed that NSCLC cells with acquired EGFR TKI resistance are more tolerant to low glucose-induced autophagy following a metabolic shift to higher glucose uptake and glycolysis activity due to upregulation of active glucose transporter SGLT1 by Thr678-phosphorylated EGFR, in a PKCδ-dependent manner. Higher SGLT1 expression correlated with poorer clinical outcomes of EGFR TKI-treated NSCLC patients while targeting SGLT1 with pharmacological inhibitors suppressed enhancements in glucose metabolism and lowered acquisition of EGFR TKI resistance in NSCLC xenograft-bearing mice. Our results not only elucidate the metabolic mechanism underlying the development of acquired resistance to EGFR TKIs but also indicate that targeting PKCδ/SGLT1 in combination with EGFR TKIs may benefit NSCLC patients.

## Results

### TKI-resistant cells are tolerant to autophagic cell death induced by glucose deprivation

**Fig. 1:**
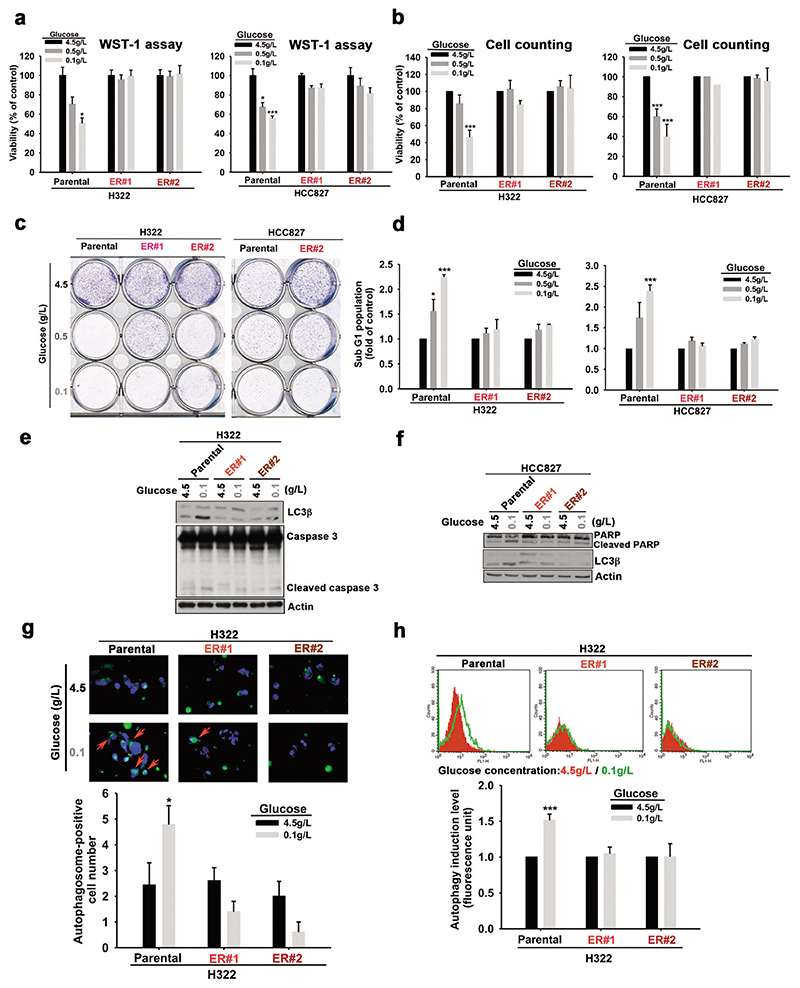
The acquired erlotinib-resistant NSCLC cells were more tolerant to glucose deprivation. H322, HCC827, and their erlotinib-resistant (ER) cells were cultured in different concentrations of glucose. **a–c** The cell viability was measured in WST-1 (**a**), cell counting (**b**), and clonogenic (**c**) assays. **d** The changes in the sub-G1 population of the indicated cells in response to different concentrations of glucose were measured with PI staining in FACS analysis. **e, f** The levels of LC3β and caspase 3 cleavage in H322, HCC827 cells and their ER clones in response to glucose deprivation were analyzed by WB. **g** The low glucose concentration-induced autophagosome accumulation was detected by staining with Cyto-ID® Green autophagy dye in fluorescence analysis was shown in the upper panel, and the number of autophagosomepositive cells was quantitated in the lower panel. **h** The effects of glucose deprivation on autophagosome formation in H322 and their ER cells were determined by FACS analysis (upper panel) and quantitated (lower panel). Data in (**a**), (**b**), (**d**), (**g**), and (**h**) represent mean ± sd from three independent experiments. *P < 0.05; ***P < 0.001 vs. control group, Student’s *t*-test. Data in (**c**), (**e**), and (**f**) are representative of three experiments.

To determine whether alternation of glucose metabolism plays a role in the development of acquired resistance to EGFR TKIs, we first established erlotinib-resistant (ER) clones from wt EGFR-expressing NSCLC lines NCI-H322 (H322) and NCI-H292 (H292) and from an activating EGFR mutant-expressing HCC827 lung adenocarcinoma cell line by culturing the cells in increasing concentrations of erlotinib (by 2.5 μM every 2–3 weeks, up to a maintenance concentration of 10 μM for 3 months). Their resistance to erlotinib was validated in WST-1 assays (Supplementary Fig. S1a–c). Interestingly, the viability of the parental cell lines, but not their corresponding ER clones, was dramatically and dose-dependently decreased by reducing glucose concentrations from 4.5 to 0.1 g/L in the culture medium in WST1 (Fig. 1a and Supplementary Fig. S1d) and cell counting assays (Fig. 1b). Similarly, ER clones of H322 and HCC827 cells were also more tolerant to glucose deprivation in clonogenic formation assays (Fig. 1c). Flow cytometric analysis further showed that glucose deprivation increased the sub-G1 population of parental H322 and HCC827 cells, but not their corresponding ER clones (Fig. 1d).

Nutrition and energy deprivation trigger autophagy in a variety of cells [32], but the roles of autophagy in determining the sensitivity to EGFR TKIs in NSCLC remain controversial [11, 33–36]. Pretreatment of H322 and HCC827 cells with 3-methyladenine (3-MA) and chloroquine (CQ), which suppress autophagic flux by targeting PtdIns3Ks [37] and autophagosome-lysosome fusion [38] respectively, prevented the suppression of viability induced by glucose starvation (Supplementary Fig. S1e), suggesting that autophagy contributes to glucose deprivation-induced cell death of these cell lines. In support of this finding, the reduction of glucose concentration induced the expression of autophagic marker (LC3β) and apoptotic markers (PARP or caspase 3 cleavages) in H322 (Fig. 1e) and HCC827 (Fig. 1f) cells, but not their corresponding ER clones. Consistently, the results from Cyto-ID^®^ Autophagy dye staining showed that glucose deprivation enhanced the numbers (Fig. 1g) and levels (Fig. 1h) of autophagosome-positive cells in the parental H322 cells, but not their ER clones. All of these findings indicated that ER NSCLC cells are more tolerant to glucose deprivation-induced autophagic cell death.

### ER lung cancer cells exhibit higher glucose uptake through SGLT1 upregulation

**Fig. 2:**
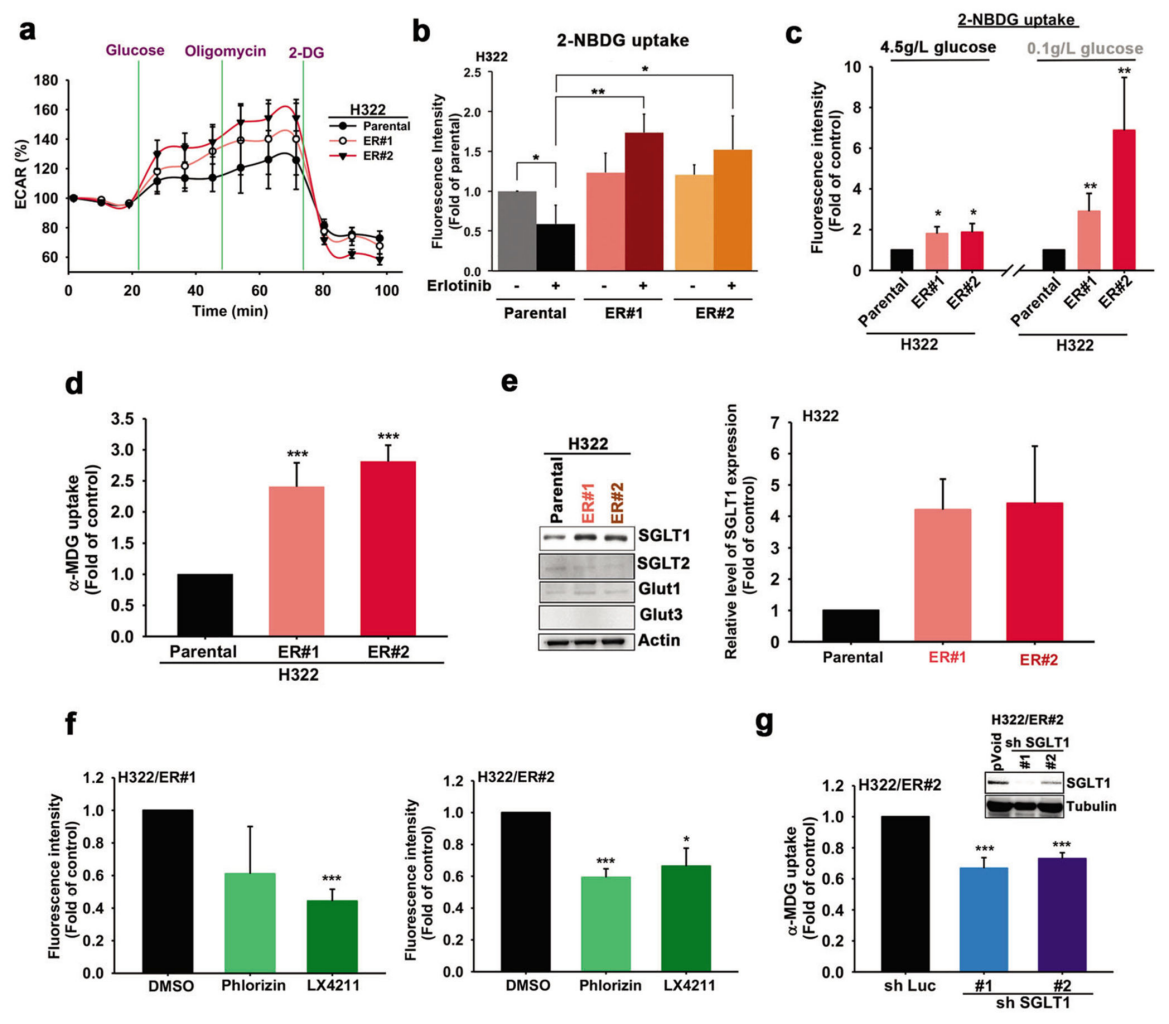
The upregulated SGLT1 mediated the glucose uptake of the acquired erlotinib-resistant cells. **a** The changes in ECAR of H322 cells and their ER clones were measured by using the XF-24 Seahorse extracellular flux analyzer. **b** 2-NBDG uptake ability of H322 cells and their ER clone was detected following EGFR-TKI-treatment. **c, d** 2-NBDG (**c**) and α-MDG (**d**) uptake ability of H322 cells and their ER clones were detected by FACS and Beckman LS6000 Scintillation Counter, respectively. **e** The protein levels of various glucose transporters in H322 cells and their ER clones were detected in WB with the indicated antibodies. **f** The effects of 100 μM phlorizin or 1 μM LX4211 on glucose uptake in the H322/ER#2 clone were measured under a low glucose concentration condition in a 2-NBDG assay. **g** The effects of SGLT1 shRNA on α-MDG uptake in the H322/ER#2 clone were measured under low glucose conditions by using the Beckman LS6000 Scintillation Counter. Data in (**b**), (**c**), (**d**), (**f**), and (**g**) represent mean ± sd from three independent experiments. *p < 0.05; **p < 0.01; ***p < 0.001 vs. control, Student’s *t*-test. Data in (**a**) and (**e**) are representative of three experiments.

Although TKI-resistant cells were more tolerant to glucose deprivation-induced cell death, inhibition of glycolysis with hexokinase inhibitor 2-deoxy-D-glucose (2-DG) still suppressed the colony formation of the corresponding ER clones of H322 and HCC827 cells (Supplementary Fig. S2), suggesting that glucose remains the critical energy source to generate ATP and cell building blocks through glycolysis for cell proliferation in these clones. The elevation in the extracellular acidification rate (ECAR), an indicator of glycolysis, in the corresponding ER clones was higher than that in the parental H322 (Fig. 2a) and HCC827 (Supplementary Fig. S3a) cells. By monitoring the engulfment of the fluorescent-labeled deoxyglucose analog 2-NBDG, the glucose uptake ability of these cells was examined. The 2-NBDG uptake was suppressed by erlotinib in H322 (Fig. 2b) and HCC827 (Supplementary Fig. S3b) cells but was higher in H322/ER and HCC827/ER clones in the presence of TKI. Interestingly, the ER#2 clone, but not its parental HCC827 cells, elicited higher ECAR under the low (0.1 g/L) glucose condition (Supplementary Fig. S3c). Increases in 2-NBDG uptake in the corresponding ER clones of H322 cells were more obvious under low glucose culture conditions than under normal glucose conditions (Fig. 2c). Active glucose transporters SGLTs are associated with 2-NBDG uptake in SGLT1-overexpressing HEK-293T cells (Supplementary Fig. S3d) consistent with previous studies [39, 40], but compared with glucose, show a much lower binding affinity with 2-DG derivatives [41]. These findings imply the involvement of SGLTs in the higher glucose uptake in these TKI-resistant clones. Indeed, the ability to increase the uptake of α-methyl-D-glucopyranoside (α-MDG), a specific substrate for SGLTs, was apparent with H322/ER (Fig. 2d) and HCC827/ER (Supplementary Fig. S3e) cells compared with their parental cells. These findings support the contention that ER cells express active glucose transporters that have a greater ability for glucose uptake.

Among cancer-related glucose transporters [42, 43], only the expression of SGLT1, not Glut1, Glut3, or SGLT2, was significantly increased in different ER clones of H322 (Fig. 2e) and HCC827 (Supplementary Fig. S3f) cells. In support of this finding, higher expression of SGLT1 in H292/ER clones than in parental cells (Supplementary Fig. S3g) was also detected by the validated anti-SGLT1 antibody (Supplementary Fig. S3h) in the xenograft tumor tissues. These data suggest that increased levels of SGLT1 may enhance glucose uptake in ER cells. Indeed, treatments with SGLT inhibitors phlorizin and LX4211 (sotagliflozin, approved in the European Union for type 1 diabetes mellitus) reduced the 2-NBDG uptake in H322/ER and HCC827/ER clones under low glucose culture conditions (Fig. 2f and Supplementary Fig. S3i) without affecting GLUT3 activity (Supplementary Fig. S3j). Similar suppressive effects of SGLT1 shRNA on α-MDG uptake were also found in different H322/ER (Fig. 2g) and HCC827/ER (Supplementary Fig. S3k) clones. SGLT1 shRNA also reduced the glucose consumption of H322/ER#1 cells (Supplementary Fig. S3l). These results suggest that upregulation of SGLT1 mediates glucose uptake and thereby supports the viability of acquired EGFR TKI-resistant cells.

### Targeting SGLT1 reduced EGFR TKI resistance in vitro and in vivo

**Fig. 3:**
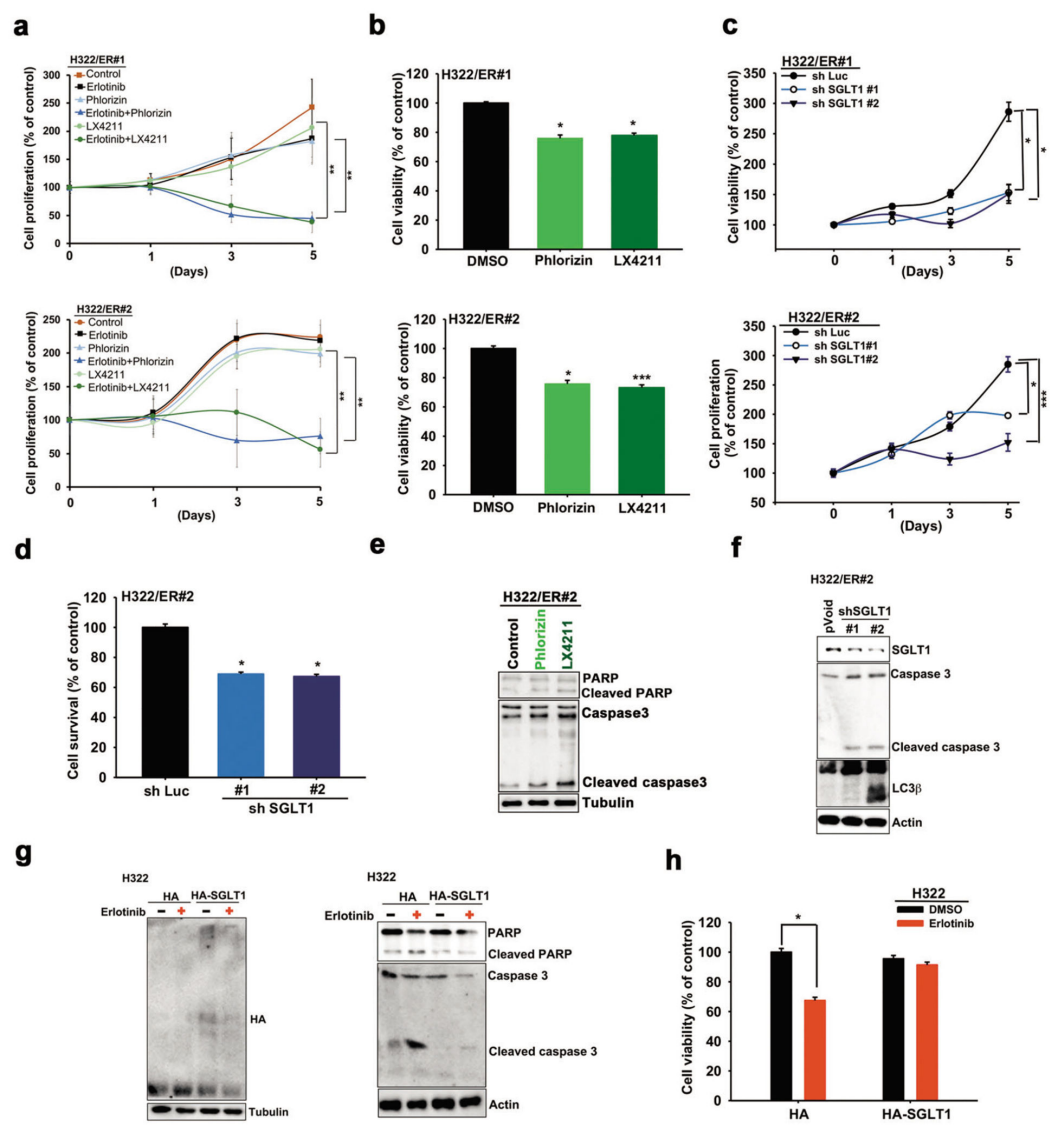
The upregulated SGLT1 supported the cell viability of the acquired TKI-resistant cells. **a** The cell proliferation of H322/ER clones in response to erlotinib, phlorizin, or LX4211 was determined in WST-1 analysis. **b** The effects of phlorizin or LX4211 on cell viability of H322/ER clones under low glucose concentration were measured in WST-1 analysis. **c, d** The effects of SGLT1 shRNA on cell proliferation (**c**) and viability (**d**) of H322/ER clones under low glucose concentration were determined by cell counting and WST-1 analyses, respectively. **e, f** The effects of SGLT1 inhibitors (**e**) and shRNA (**f**) on caspase 3 or PARP cleavages or LC3β in H322/ER clones were analyzed by WB. **g, h** The effects of SGLT1 overexpression on the erlotinib-induced PARP and caspase 3 cleavages (**g**) and cell death (**h**) in H322 cells were examined in WB and WST-1 analyses, respectively. Data in (**a**)–(**d**), and (**h**) represent mean and sd from three independent experiments. *p < 0.05; **p < 0.01; ***p < 0.001 vs. control, Student’s *t*-test. Data in (e)–(g) are representative of three experiments.

We next examined the role of SGLT1 in conferring drug resistance to EGFR TKIs through increasing glucose uptake. Treatments with phlorizin or LX4211 decreased the proliferation rate of ER clones of H322 (Fig. 3a) and HCC827 (Supplementary Fig. S4a) cells and their viability under low glucose culture conditions (Fig. 3b and Supplementary Fig. S4b). Consistently, shRNA-mediated depletion of SGLT1 limited the cell growth of different H322/ER (Fig. 3c) and HCC827/ER clones (Supplementary Fig. S4c), and re-sensitized these ER clones to cell death induced by glucose deprivation (Fig. 3d and Supplementary Fig. S4d). Treatments with SGLT1 inhibitors phlorizin or LX4211 (Fig. 3e, Supplementary Fig. S4e) or shRNA (Fig. 3f and Supplementary Fig. S4f) also enhanced the glucose deprivation-induced caspase 3 or PARP cleavage and LC3β in H322/ER#2 and HCC827/ER#2 cells, but not in the parental H322 cells (Supplementary Fig. S4g). Conversely, overexpression of SGLT1 was not only associated with higher α-MDG uptake (Supplementary Fig. S4h) and lower levels of apoptotic markers (Supplementary Fig. S4i) and cell death (Supplementary Fig. S4j) in response to glucose deprivation in H322 cells, but SLGT1 overexpression also attenuated erlotinib-induced PARP and caspase 3 cleavages in parental H322 cells (Fig. 3g) and HCC827 cells (Supplementary Fig. S4k) and consequently restored the TKI-suppressed viability (Fig. 3h and Supplementary Fig. S4l). These results support the contention that increased SGLT1 expression enhances glucose uptake and thereby reduces the sensitivity of NSCLC cells to EGFR TKIs.

**Fig. 4:**
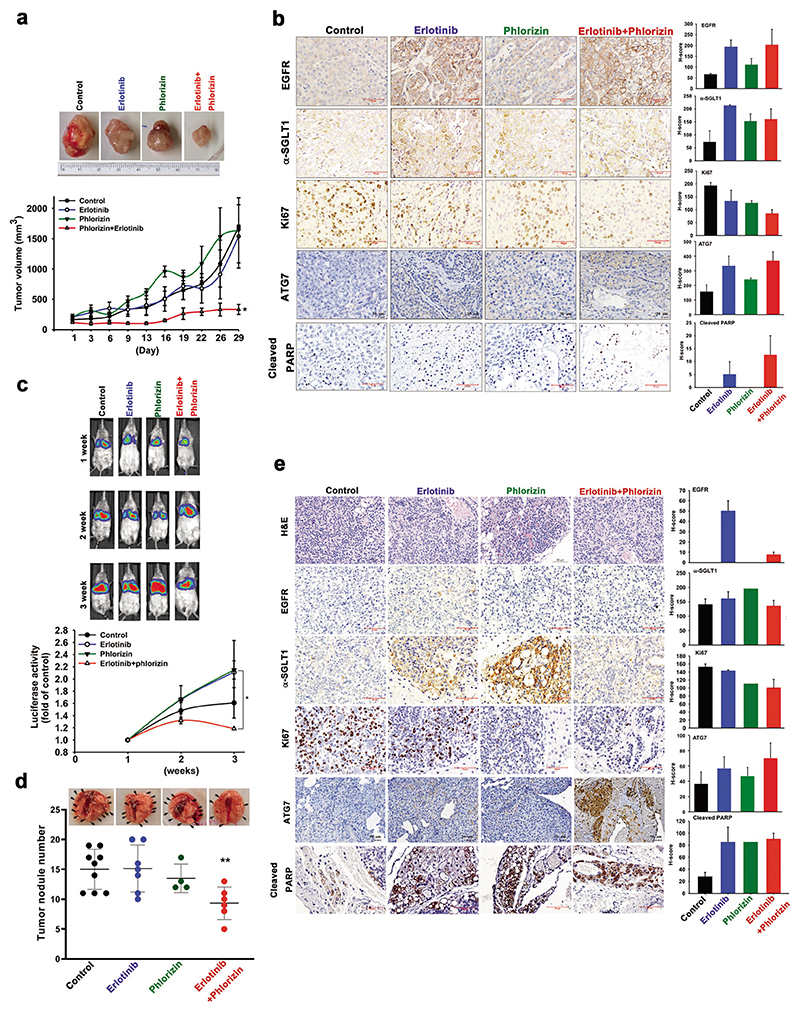
Targeting SGLT1 reduced the development of acquired resistance to EGFR TKI in vivo. **a** The growth rate of xenograft tumors of H292 cells in response to treatments with erlotinib, phlorizin, or the combination was determined by measuring tumor size. **b** The representative IHC staining of tumor sections from (**a**) were shown (left) and the results from five independent sections for all groups were quantitated with H-score (Right). Scale bar, 50 μm. **c–e** SCID mice injected with A549-Luc cells were treated with erlotinib, phlorizin, or the combination. The tumor volumes were measured by detecting luminescent signals in the lumina LT in vivo imaging system (upper in panel (**c**)) and the luciferase activity was quantitated (lower in panel (**c**)). After treatment for 3 weeks, the lungs of A549 cell-xenograft SCID mice were harvested and the numbers of lung tumor nodules were quantitated. e The representative IHC staining of tumor sections from (**d**) was shown (left) and the results from five independent sections for all groups were quantitated with H-score (Right). Scale bar, 50 μm. Data represent mean ± sd *p < 0.05 and **p < 0.01 vs. control, Student’s *t*-test.

Next, we addressed whether SGLT1 inhibitors can enhance the therapeutic efficacy of EGFR TKIs in tumor xenograft mouse models. First, SCID mice bearing H292 subcutaneous xenografts were treated with erlotinib (50 mg/kg), phlorizin (20 mg/kg), LX4211 (60 mg/kg) alone, or the combination of erlotinib with either phlorizin or LX4211. Treatments with erlotinib, phlorizin, or LX4211 alone did not significantly reduce tumor growth, but the combination of erlotinib with either phlorizin (Fig. 4a) or LX4211 (Supplementary Fig. S5a) significantly suppressed the tumor growth. After treatment with erlotinib for 29 days, levels of EGFR and SGLT1 expression were elevated in the xenograft tumor tissues in IHC staining analysis. The combination treatments also enhanced the reduction of the proliferation marker Ki67 and the induction of apoptosis marker PARP cleavage and ATG7, which is induced to mediate autophagy under glucose deprivation [44], in the xenograft tumor tissues (Fig. 4b and Supplementary Fig. S5b). To examine whether SGLT1 also plays a role in conferring intrinsic resistance to EGFR TKIs, the experimental metastatic NSCLC xenograft model was established by tail vein injection of SGLT1-positive and TKI-insensitive A549 cells transfected with the luciferase gene. Co-treatment with erlotinib and phlorizin reduced the tumor size (Fig. 4c) and the number of tumor nodules (Fig. 4d). Remarkable reductions in Ki67 expression, as well as increases in ATG7 expression and PARP cleavage, were also found in the lung tumor tissues after co-treatment with erlotinib and phlorizin (Fig. 4e). Together, these findings support the therapeutic potential of SGLT1 inhibitors for reducing the development of acquired EGFR TKI resistance in NSCLC patients.

### SGLT1 is upregulated in recurrent tumor tissues after the failure of EGFR TKI treatment and is associated with a poor prognosis of NSCLC

**Fig. 5:**
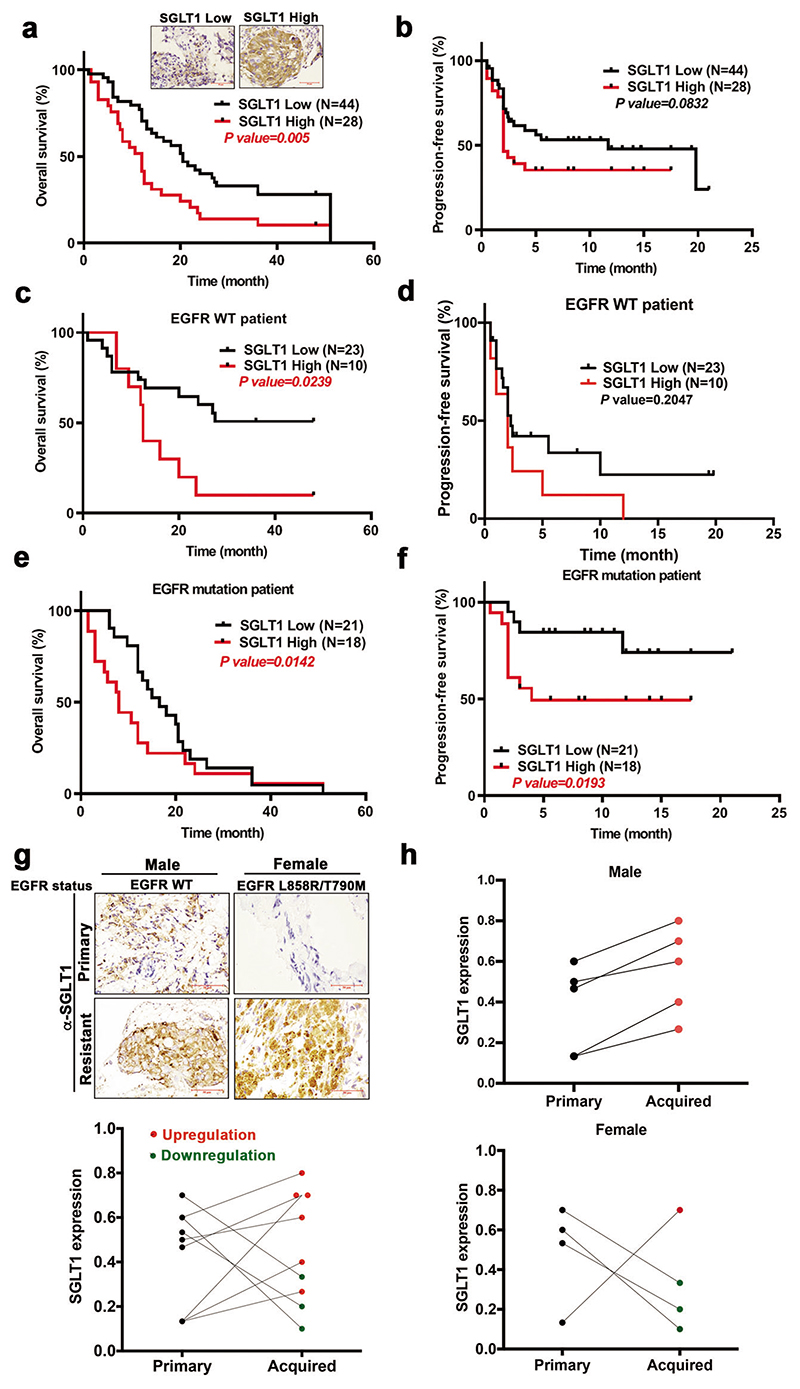
SGLT1 expression negatively correlates with the clinical benefits of EGFR TKI in NSCLC patients. **a, b** Tumor tissues from total NSCLC patients ever treated with EGFR TKIs were subjected to IHC staining with anti-SGLT1 antibody ((**a**) upper). Scale bar, 50 μm. The clinical correlation of SGLT1 expression with overall survival (**a**) and progression-free survival (PFS) (**b**) rates were analyzed in Kaplan–Meier analysis. **c–f** The EGFR TKI-treated NSCLC patients in (a and b) were further classified into wt EGFR (**c**) and (**d**) and mutant EGFR (**e**) and (**f**) groups for Kaplan–Meier overall survival and progression-free survival analysis. **g, h** SGLT1 protein levels in the paired tissues from treatment-naïve tumors and acquired TKI-resistant tumors of nine lung cancer patients were examined by IHC staining (upper in panel (**g**)) and quantitated (lower in panel (**g**)). The results from panel (**g**) were further divided into two groups according to the genders of patients (**h**). Scale bar, 50 μm.

In the Kaplan–Meier survival analysis of records from the public transcriptomic database [45], higher SGLT1 mRNA expression correlated with an increasingly worse overall survival rate in lung adenocarcinoma patients (Supplementary Fig. S6a), which was reflected in both smoker and non-smoker populations (Supplementary Fig. S6b, c). We next analyzed the clinical correlation of SGLT1 expression with prognosis in 72 NSCLC patients who carried either wt or mutant EGFR and received erlotinib or gefitinib treatments. SGLT1 expression was associated with gender and age, but not smoking behavior, EGFR mutation status, tumor size, lymph node metastasis, pathological stage, or immediate response to EGFR TKIs, with higher SGLT1 expression, detected in NSCLC tumors of males aged over 55 years (Supplementary Table S1 and Supplementary Fig. S6d, e). EGFR TKI-treated NSCLC patients with the higher SGLT1-expressing tumors showed a lower overall survival rate with statistical significance (Fig. 5a) and a more unfavorable progression-free survival rate with marginal significance (Fig. 5b). The negative correlations of SGLT1 expression with overall and progression-free survival rates were also observed in both NSCLC subpopulations; in patients with wt EGFR tumors (Fig. 5c, d) and those with EGFR mutations (Fig. 5e, f), respectively.

We then examined the change in SGLT1 expression in human NSCLC tumor tissues after the development of acquired resistance to EGFR TKIs. Treatment-naïve primary tumor tissues paired with the acquired EGFR TKI-resistant tumor tissues were collected from 9 NSCLC patients and were subjected to IHC staining with an anti-SGLT1 antibody. In response to acquired EGFR TKI resistance, recurrent tumor tissues from 6 of 9 patients demonstrated upregulated SGLT1 expression (Fig. 5g). Interestingly, SGLT1 expression was increased in all 5 male patients but was decreased in 3 of the 4 female patients (Fig. 5h). These results suggest that SGLT1 upregulation may contribute to the acquired resistance to EGFR TKIs in NSCLC patients, especially in males. It is not yet clear whether hormone receptors are involved in the regulation of SGLT1 expression in lung cancer tissues and would be worthy of further investigation.

### Elevated EGFR expression stabilizes SGLT1 expression to support the survival of TKI-resistant cells

**Fig. 6:**
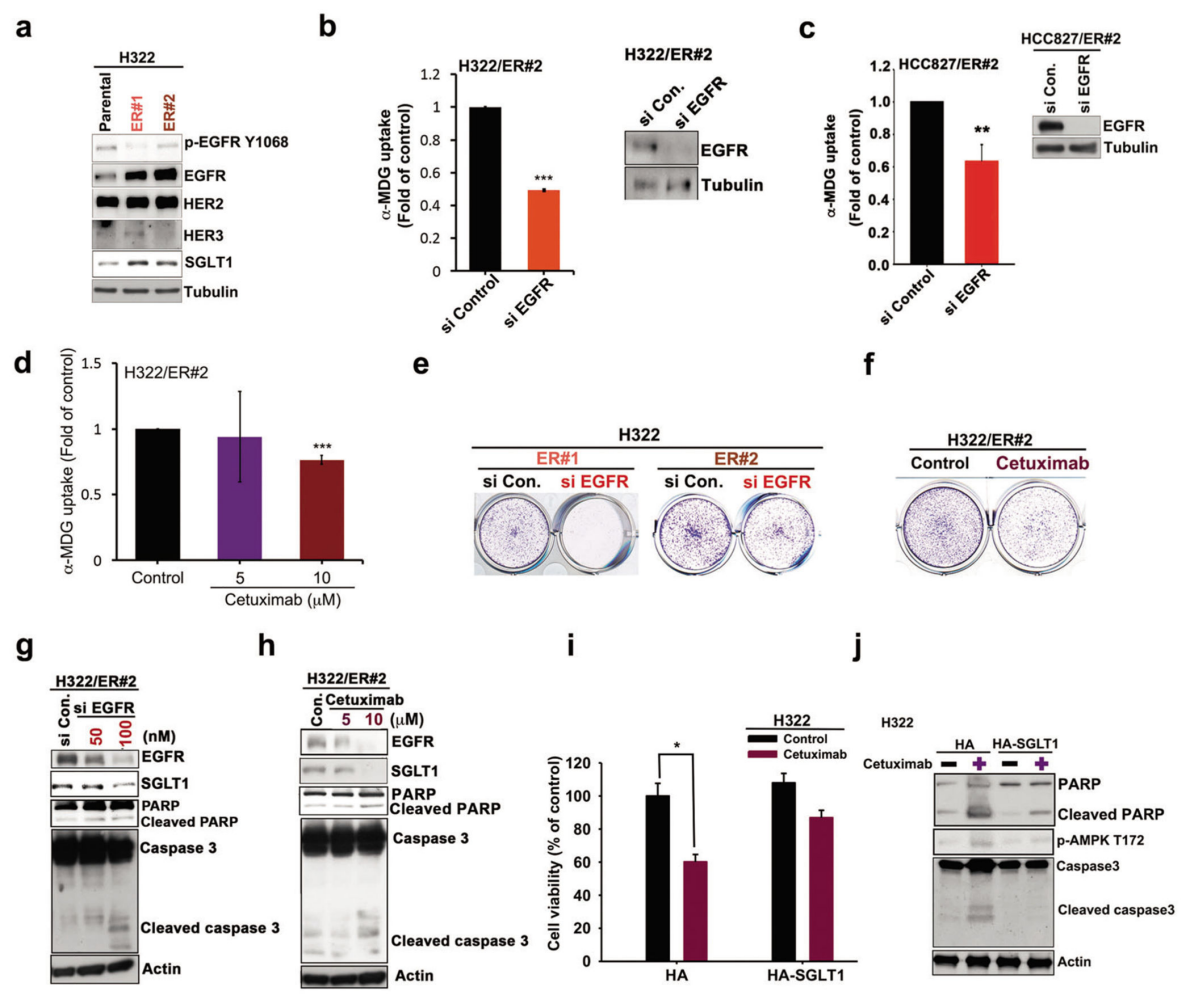
The increased EGFR mediated glucose uptake and viability of the acquired erlotinib-resistant cells through SGLT1 upregulation. **a** The protein expressions of the ErbB family and SGLT1 in H322 cells and their ER clones were analyzed in WB analysis. **b**–**h** The effects of EGFR siRNA or monoclonal antibody cetuximab on α-MDG uptake (**b**)–(**d**), colony formation (**e**) and (**f**), and caspase and PARP cleavages (**g**) and (**h**) of H322/ER#2 and HCC827/ER#2 cells were examined, respectively. **i, j** The effects of SGLT1 over expression on the cetuximab-induced viability inhibition (**i**) or PARP and caspase 3 cleavages (**j**) of H322 cells were examined in WST-1 analysis and WB, respectively. Data in (**c**) and (**d**) and (**i**) represent mean ± sd from three independent experiments. *p < 0.05; ***p < 0.001 vs. control, Student’s *t*-test. Data in (**a**), (**e**)–(**h**), and (**j**) are representative of three experiments.

EGFR reportedly stabilizes SGLT1 expression, independent of its tyrosine kinase activity [46]. We observed elevations in EGFR expression after treatment with erlotinib (Fig. 4b and Supplementary Fig. S5b). Thus, we sought to determine whether the upregulation of SGLT1 in TKI-resistant cells depends upon EGFR. We observed increases in both SGLT1 expression and EGFR protein levels, but also a decrease in EGFR Y1068 phosphorylation, a marker for EGFR tyrosine kinase activity, in the TKI-resistant H322 clones in the presence of erlotinib (Fig. 6a). Similarly, EGFR upregulation was also observed in the H292/ER xenograft tumor tissues compared to that of their parental cells in SCID mice (Supplementary Fig. S7a). Silencing EGFR expression with specific siRNAs reduced the uptake of α-MDG (Fig. 6b, c) and 2-NBDG (Supplementary Fig. S7b) in ER clones. Similarly, downregulation of EGFR by treatment with cetuximab, the EGFR monoclonal antibody, suppressed α-MDG (Fig. 6d) and 2-NBDG (Supplementary Fig. S7c) uptake in ER clones. Treatments with EGFR siRNAs (Fig. 6e and Supplementary Fig. S7d) or cetuximab (Fig. 6f and Supplementary Fig. S7e) also reduced the cell growth of H322/ER and HCC827/ER clones in colony formation assays. Moreover, downregulation of EGFR by siRNA administration (Fig. 6g and Supplementary Fig. S7f) or cetuximab (Fig. 6h and Supplementary Fig. S7g) reduced both EGFR and SGLT1 protein levels and also increased PARP and caspase 3 cleavages in H322/ER and HCC827/ER clones. In contrast, overexpression of SGLT1 attenuated cetuximab-induced cell death (Fig. 6i and Supplementary Fig. S7h), PARP and caspase 3 cleavage, and AMPK activation (Fig. 6j) in parental NSCLC cells. These results support the notion that EGFR, in the presence of TKIs, upregulates SGLT1 expression to support cell growth and survival in cells with acquired EGFR TKI resistance.

### Phosphorylation of EGFR T678 by PKCδ mediates SGLT1 protein stabilization by enhancing the interaction between EGFR and SGLT1

**Fig. 7:**
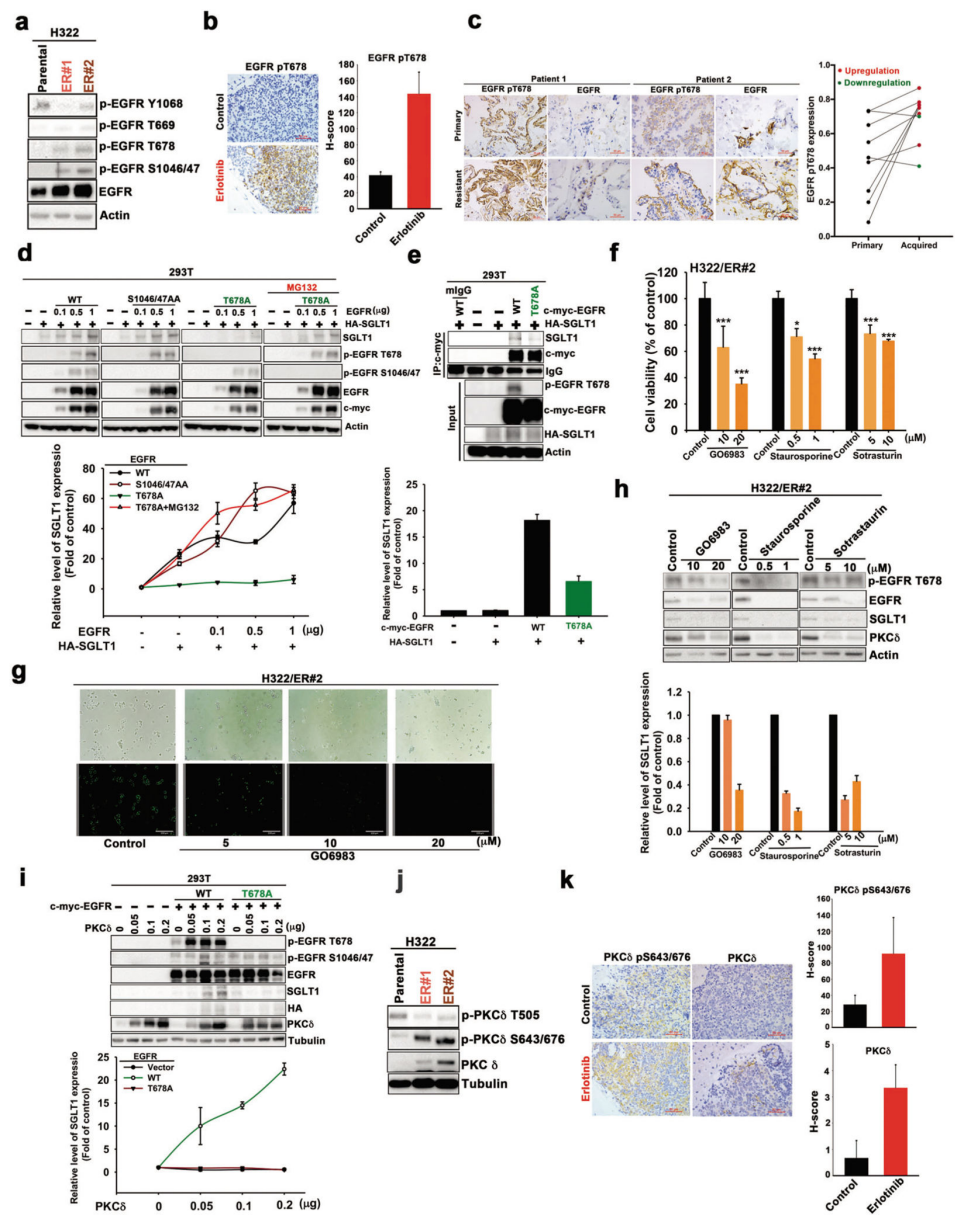
EGFR Thr678 phosphorylation by PKC delta mediated the SGLT1/EGFR interaction for SGLT1 protein stabilization. **a** EGFR phosphorylations in H322 cells and their ER clones were examined in WB with the indicated antibodies. **b** EGFR pT678 phosphorylation in the expression of IHC pictures in lung sections (left) and the H-score of protein expression was shown (right). Scale bar, 50 μm. **c** EGFR T678 phosphorylation and EGFR protein level in the paired treatment-naïve and acquired TKI-resistant tumor tissues of lung cancer patients was determined by IHC staining. Scale bar, 50 μm. **d, e** The indicated protein expression in HEK-293T cells co-transfected with EGFR mutants and SGLT1 in the presence or absence of MG-132 was analyzed by WB (**d**) and IP/WB (**e**). **f** The effects of GO6983, staurosporine, or sotrasturin on cell viability were determined in WST-1 assay. **g** Glucose uptake in H322/ER cells in response to GO6983 treatment was analyzed with the 2-NBDG uptake assay. Scale bar, 330 μm. **h–j** The total lysates from H322/ER#2 cells treated with various PKC inhibitors (**h**), HEK-293T cells co-transfected with EGFR mutants, and PKCδ (**i**), and H322 cells and ER clones (**h**) were subjected to WB with the indicated antibodies. **k** PKCδ pS643/676 and PKCδ expression in the H292 cells-xenograft tumor tissues in response to erlotinib treatment were examined in IHC analysis (left panel), and the H-score of protein expression were shown (right). Scale bar, 50 μm. Data are shown in (**a**) and (**d**)–(**j**) represent mean and s. d. from three experiments. Data in (**b**) and (**k**) are representative of five experiments and shown as mean ± sd. *p < 0.05; **p < 0.01; ***p < 0.001 vs. control group, Student’s *t*-test.

We next investigated how EGFR stabilizes SGLT1 in TKI-resistant cells independent of its tyrosine kinase activity. Despite tyrosine autophosphorylation, serine or threonine phosphorylation in members of the ErbB family has been found to regulate their kinase activity, protein stability, or endocytosis [47, 48]. Interestingly, we observed that EGFR phosphorylations at T678 and Ser1046/1047, but not T669, all of which are mediated by different Ser/Thr kinases [49–52], were increased in H322/ER clones while Y1068 phosphorylation was suppressed by erlotinib (Fig. 7a and Supplementary Fig. S8a). The upregulation of EGFR T678 phosphorylation was also observed in the xenograft tumor tissue of parental H292 cells after 1 month of erlotinib treatment (Fig. 7b), in the xenograft tissues of H292/ER clones (Supplementary Fig. S8b), and in human NSCLC tumors with acquired TKI resistance (Fig. 7c), compared with their parental counterparts. Mutation of T678, but not S1046/1047, to Ala abolished EGFR-enhanced SGLT1 expression, and this effect was reversed by treatment with proteasome inhibitor MG132 in HEK-293T cells (Fig. 7d). Mutation of T678A also reduced the interaction between EGFR and SGLT1 (Fig. 7e). These results suggest that increased T678 phosphorylation of EGFR mediates SGLT1 protein stabilization through protein–protein interaction.

PKC has been reported to phosphorylate EGFR at T678 to protect EGFR from degradation [49, 50]. Protein kinase Cδ has been found to contribute to the acquired resistance of NSCLCs to EGFR TKIs [12], but the underlying mechanisms remain unclear. Therefore, we next asked whether PKCδ is involved in SGLT1-mediated TKI resistance through EGFR T678 phosphorylation. The viabilities of H322/ER#2 (Fig. 7f) and HCC827/ER#2 (Supplementary Fig. S8c) cells were decreased by treatment with the pan-PKC inhibitors GO6983, staurosporine, and sotrasturin, in a dose-dependent manner. GO6983 also suppressed basal and glucose-induced ECAR (Supplementary Fig. S8d, e) and glucose uptake (Fig. 7g and Supplementary Fig. S8f) in H322/ER#2 and HCC827/ER#2 cells. These PKC inhibitors also suppressed EGFR T678 phosphorylation and EGFR and SGLT1 protein levels (Fig. 7h and Supplementary Fig. S8g). Overexpression of PKCδ induced increases in EGFR levels, EGFR T678 phosphorylation, and SGLT1 protein levels in H322 cells (Supplementary Fig. S8h), and further enhanced EGFR-mediated SGLT1 upregulation, which was abolished by the mutation of EGFR T678A (Fig. 7i). These findings indicate that PKCδ, in addition to its nuclear functions [12], confers EGFR TKI resistance by regulating the formation of the EGFR/SGLT1 complex in the cytoplasm.

Interestingly, PKCδ S643/676 phosphorylation, which is mediated by mTORC2, but not T505 phosphorylation, was observed in the H322/ER and HCC827/ER clones (Fig. 7j and Supplementary Fig. S8i) and in the xenograft tumor tissues of parental H292 cells after 1 month of erlotinib treatment (Fig. 7k). Since mTORC2 is inactivated by S6K in response to the signaling of the EGFR/mTORC1 axis [53], the mTORC2/PKCδ axis may be activated and mediate EGFR T678 phosphorylation for SGLT1 protein stabilization, when EGFR tyrosine kinase activity was suppressed by TKIs. In support of this notion, the mTORC1/2 inhibitor everolimus suppressed not only PKCδ S643/676 phosphorylation, but also EGFR T678 phosphorylation and SGLT1 levels (Supplementary Fig. S8j).

**Fig. 8:**
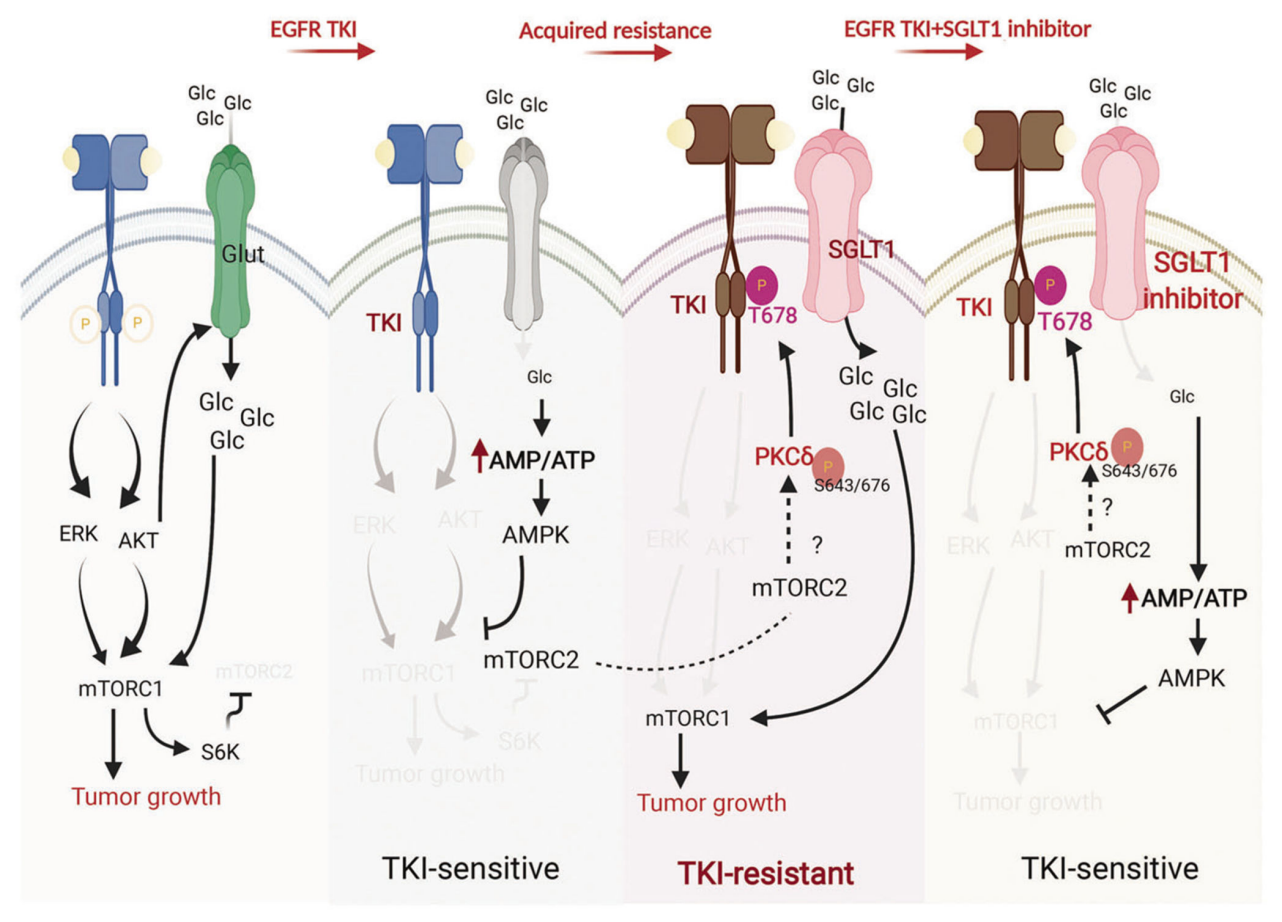
A proposed model illustrating the involvement of SGLT1-mediated metabolic reprogramming in the development of acquired resistance to EGFR TKIs. In sensitive NSCLC cells, EGFR TKIs suppressed EGFR and its downstream Akt and ERK signaling pathways as well as the membrane level of GLUT and glucose uptake. Reduced glucose uptake leads to lower ATP production, an increase in the intracellular AMP/ATP ratio, and activation of AMPK for the suppression of mTORC1 and tumor growth. In the presence of TKIs, however, PKCδ is activated by mTORC2 to phosphorylate EGFR at T678 to stabilize SGLT1 protein in an EGFR tyrosine kinase-independent manner while EGFR/mTORC1/S6K axis was inhibited. Even under a low glucose environment, the elevated SGLT1 engulfs more glucose to maintain intracellular glucose level and subsequently ATP production for cell survival, leading to the acquired EGFR TKI resistance. Co-treatment with SGLT1 inhibitors may avoid the development of acquired EGFR TKI resistance.

In conclusion, TKIs suppressed EGFR and its downstream Akt and ERK signaling pathways, as well as the membrane levels of GLUT and glucose uptake in the sensitive cells. Reduced glucose uptake led to a subsequent reduction in ATP production, an increase in the intracellular AMP/ATP ratio, and activation of AMPK for the suppression of mTORC1 and tumor growth (Fig. 8, left). However, while EGFR tyrosine kinase activity was suppressed by long-term TKI treatment, mTORC2 was activated from the EGFR/mTORC1/S6K axis to phosphorylate PKCδ for subsequent EGFR T678 phosphorylation and SGLT1 protein stabilization. Even in an environment of low glucose, higher SGLT1 expression engulfs more glucose to maintain intracellular glucose levels, leading to acquired EGFR TKI resistance and maintenance of ATP production for cell viability (Fig. 8, right). Thus, co-treatment with SGLT1 inhibitors may avoid the development of acquired resistance to EGFR TKIs.

## Discussion

In addition to the inhibition of EGFR downstream PI3K/Akt and MAPK survival pathways, reductions in glucose uptake and glycolysis have been detected in gefitinib-treated lung cancer cells that precede cell cycle suppression and apoptosis induction [22], suggesting that glucose metabolic activity closely reflects the intrinsic response to EGFR TKI-based therapy. However, it remains unclear as to whether the development of acquired resistance to EGFR TKIs involves the reprogramming of glucose metabolism in NSCLC cells. In this study, our results showed that protein stability of the active glucose transporter SGLT1 was dramatically enhanced by EGFR relying on PKCδ-mediated phosphorylation to support glucose uptake and viability of ER lung cancer cells.

Cancer cells support their growth by expressing glucose transporters that increase glucose uptake from the extracellular environment. Treatment with TKIs decreases glucose consumption and lactate production by inhibiting the translocation of the Glut3 transporter from the cytosol to the plasma membrane in lung adenocarcinoma [24]. The downregulation of Glut1 and Glut3 protein levels also accounts for the anticancer activity of EGFR TKIs in NSCLC cell lines and xenograft tumor tissues [24, 25]. EGFR expression and downstream signaling pathways have shown positive correlations with Glut1 expression and membrane localization in lung cancer [54], pancreatic cancer tissue [55, 56], and triple-negative breast cancer (TNBC) [57], suggesting that TKI-sensitive cancer cells employ passive glucose transporters to engulf glucose and that downregulation of these transporters may account for the anticancer activity of TKIs. Under adaptation to long-term TKI treatment, however, SGLT1 upregulation compensated for glucose uptake in TKI-resistant cells in our study.

SGLT1 is generally expressed in the normal epithelial cells of the small intestine to transport glucose and galactose across the luminal side of enterocytes, but its overexpression in prostate cancer [58] and colorectal cancer [59] is associated with poor prognosis. Although SGLT2, typically expressed in the renal proximal tubule, has also been reported to play a critical role in the development of pancreatic or breast cancer [60, 61], our data showed that SGLT1, but not SGLT2, is upregulated in response to EGFR TKI treatment, which supports glucose uptake and cellular viability in NSCLC cells with acquired TKI resistance. Although the EGF-activated PI3K/Akt/CREB signaling axis upregulates SGLT1 expression and enhances glucose uptake in intestine epithelial cells [62], the tyrosine kinase activity of EGFR or IGF1R and Akt signals are not needed for SGLT1 upregulation in cancer cells [46, 63]. In this study, our data showed that enhanced SGLT1 protein expression and glucose uptake in TKI-resistant cells were mediated by kinase-inactive EGFR (Fig. 6). Downregulation of EGFR expression involves the direct targeting of its 3′UTR activity by microRNA-7 [64–67]. Interestingly, the kinase-dependent functioning of EGFR has been reported to induce miR-7 transcription [68], suggesting that miR-7 acts as a negative feedback regulator of EGFR expression. Indeed, EGFR expression without tyrosine kinase activity is increased in response to TKI treatment, which down-regulates microRNA-7 and promotes the migration and invasion of TNBC cells [64, 69]. This negative feedback regulation of EGFR may also contribute to the protein stabilization of SGLT1 in NSCLC cells with acquired EGFR TKI resistance.

Besides the elevation of EGFR protein levels, our data revealed that PKCδ-mediated EGFR T678 phosphorylation also enhanced the protein–protein interaction of EGFR with SGLT1 to stabilize SGLT1 protein levels in TKI-resistant cells (Fig. 7). This conserved phosphorylation by PKC reduces endocytic trafficking of EGFR and ErbB3 [70, 71], and the retention of these receptors on the plasma membrane may account for the higher interaction between EGFR and SGLT1 in TKI-resistant cells. Moreover, our data found that mTORC2-mediated PKCδ phosphorylation (Ser643 and Ser676) was notably increased in the cells with acquired resistance to erlotinib and that mTORC1/2 inhibition suppressed PKCδ activation and EGFR/SGLT1 protein expression. PKCδ-mediated EGFR/SGLT1 stabilization may be involved in the mTORC2-mediated metabolic reprogramming in EGFR TKI-resistant NSCLC cells [72]. These findings also support targeting of mTORC2, which is activated due to the inhibition of EGFR/mTORC1/S6K signaling [53], as another therapeutic strategy to overcome EGFR TKI resistance in NSCLC cells [72].

Since SGLTs are functionally active in various cancer types, including pancreatic, prostate, and brain cancers [60], the use of new antidiabetic SGLT inhibitors for cancer therapy has also emerged as a possibility [73]. Four SGLT2 inhibitors, empagliflozin (Jardiance), dapagliflozin (Forxiga), canagliflozin (Invokana), and ertugliflozin (Stelagro) have recently been approved for the treatment of type 2 diabetes mellitus by the US Food and Drug Administration, and are associated with beneficial effects in the cardiovascular system and the kidney [74]. Randomized clinical trial data have not revealed any associations between treatment with these SGLT2 inhibitors and increased incidence rates of malignancies [75]. Our data show that co-targeting SGLT1 with LX4211 (sotagliflozin) enhanced the anticancer activity of EGFR TKIs in mouse models. LX4211 is an orally-delivered dual SGLT1/2 inhibitor approved for T1DM and exhibits a 20-fold higher potency for SGLT2 over SGLT1 [76]; the involvement of SGLT2 inhibition in this synergism cannot be ruled out. Moreover, this dual inhibitor improves severe glycemic and non-glycemic outcomes without severe gastrointestinal side effects [77], although its associated increased risk of diabetic ketoacidosis in patients with type 1 diabetes mellitus is of concern [78]. The possibility of this event should be evaluated during clinical testing of the synergistic effects of LX4211 on the anticancer activity of EGFR TKIs.

In conclusion, our results elucidate the role of PKCδ/EGFR-dependent SGLT1 expression in the rewiring of glucose uptake and metabolism that supports the expansion of cells with acquired TKI resistance (Fig. 8). These findings indicate that SGLT1 is a potential target for avoiding acquired resistance to EGFR TKI therapy in NSCLC.

## Supplementary Material

**Supplementary information** The online version contains supplementary material available at https://doi.org/10.1038/s41388-021-01889-0.

Supplementary Data
