# Supplementary material for: PKCδ-mediated SGLT1 upregulation confers the acquired resistance of NSCLC to EGFR TKIs
Source: Oncogene. Author manuscript; Available in PMC 2021 Jul 29. (PMC8298203; doi:10.1038/s41388-021-01889-0)
Supplement: Supplementary Data [file EMS127164-supplement-Supplementary_Data.pdf]

Supporting Information

PKCδ-mediated SGLT1 upregulation confers the acquired resistance of NSCLC to EGFR TKIs

Chia-Hung Chen, Bo-Wei Wang, Yu-Chun Hsiao, Chun-Yi Wu, Fang-Ju Cheng, Te-Chun Hsia, Chih-Yi Chen, Yihua Wang, Zhang Weihua, Ruey-Hwang Chou, Chih-Hsin Tang, Yun-Ju Chen, Ya-Ling Wei, Jennifer L. Hsu, Chih-Yen Tu, Mien-Chie Hung, Wei-Chien Huang

This file includes:

Materials and Methods

Supplementary Fig 1~8

Supplementary Table 1

<u>Materials and Methods</u>

Reagents and plasmids

Erlotinib (#S1023) and gefitinib (#S5098) were obtained from Selleckchem (Houston, TX, USA). SGLT inhibitors phlorizin (# 60-81-1) and LX4211 (# HY-15516) were obtained from PubChem (Bethesda, MD, USA) and MedChem Express (Monmouth Junction, NJ, USA), respectively. D-glucose (#15023-021) was purchased from Thermo Fisher Scientific (Waltham, MA, USA). 2-Deoxy-D-glucose (2-DG) (#MER-25972), 3-methyladenine (3-MA) (#SI-M9281), chloroquine (CQ) (# SI-C6628), propidium iodide (PI) (#P4170), and oligomycin (# 75351) were purchased from Sigma-Aldrich. MG-132 (# 10012628), GO6983 (# 13311), sotrastaurin (#16726), staurosporine (#81590), and everolimus (#11597) were purchased from Cayman Chemical (Ann Arbor, Michigan, USA). HBDDE (#sc-202174) was obtained from Santa Cruz Biotechnology. pLKO-shSGLT1#1(TRCN0000043590), pLKO-shSGLT1#2 (TRCN0000043592), pCMV-ΔR8.91, and pMD.G were purchased from the National RNAi Core Facility of Academia Sinica (Taipei, Taiwan).

The rabbit polyclonal antibodies specific for SGLT1 was generated from LTK BioLaboratories (Taoyuan, Taiwan). The rabbit polyclonal antibodies against EGFR (#sc-03; RRID:AB_631420), HER2 (Neu) (#sc-393712; RRID:AB_2810840), HER3 (#sc-7390; RRID:AB_2262346), HER4 (#sc-283; RRID:AB_2231308), Glut1 (#sc-7903; RRID:AB_2190936) were purchased from Santa Cruz Biotechnology (Dallas, Texas, USA). The rabbit polyclonal antibodies specific for PARP (#9542; RRID:AB_2160739), cleaved PARP (#5625; RRID:AB_10699459), phospho-EGFR S1046/47 (#2238; RRID:AB_331129), phospho-EGFR T669 (#3056; RRID:AB_1264152), phospho-EGFR T678 (#14343; RRID:AB_2798457),

phospho-PKC δ S643/676 (#9376; RRID:AB_2168834), phospho-AMPK T172 (#2531; RRID:AB_330330), phospho-mTOR S2448 (#2971; RRID:AB_330970), mTOR (#2983; RRID:AB_2105622) and caspase 3 (#9662; RRID:AB_331439) were from Cell Signaling Technology (Danvers, MA, USA). LC3 (#NB100-2220SS; RRID:AB_791015) antibody was acquired from Novus Biologicals (Centennial CO, USA). Rabbit polyclonal antibodies specific for PKC δ (#ab182126) and phospho-PKCδ T505 (# ab60992; RRID:AB_944848) were obtained from Abcam (Cambridge, United Kingdom, England). Mouse polyclonal antibody specific for phospho-EGFR Y1068 (#2236; RRID:AB_331792) was purchased from Cell Signaling Technology. Mouse polyclonal antibody specific for Glut3 (sc-74497; RRID:AB_1124974) was acquired from Santa Cruz Biotechnology. The specific rabbit polyclonal antibodies of IHC staining for SGLT1 (#ab14685; RRID:AB_301410) and phospho-EGFR T678 (#ab194733) were purchased from Abcam. Goat polyclonal antibody specific of SGLT2 (#sc-47402; RRID:AB_2189561) was obtained from Santa Cruz Biotechnology. Actin (#A2228; RRID:AB_476697) and Tubulin (#T5168; RRID:AB_477579) were acquired from Sigma-Aldrich (St. Louis, Missouri, USA). HA (#11583816001; RRID:AB_514505) and Ki67 (#RM-9106; RRID:AB_2341197) were purchased from Roche (Basel, Switzerland) and Thermo Fisher Scientific (Waltham, MA, USA), respectively.

Cell culture and establishment of erlotinib-resistant (ER) clones

Human lung cancer H322 (CRL-5806; RRID:CVCL_1556), H292 (CRL1848; RRID:CVCL_0455), A549/Luc, and HCC827 (CRL-2868; RRID:CVCL_2063) cell lines and their erlotinib-resistant (ER) derivatives were cultured in RPMI 1640 medium supplemented with 10% FBS, 100 U/mL penicillin, and 100 mg/mL

streptomycin, with 10 mM HEPES. All cancer cell lines were maintained in a humidified 5% CO_2 incubator at 37 °C. The ER clones of various lung cancer cell lines were established from the parental cells by chronic treatment with gradually increasing concentrations (up to 1μM) of erlotinib.

Cell counting and cell viability assays

Cell viability was carried out in WST-1 colorimetric assays. Briefly, cells seeded in 96-well plates were pretreated with indicated inhibitors or infected with viral shRNA for 24 or 72 hrs followed by the incubation with 10μl/well of WST-1 (Roche, Basel, Switzerland) reagent to the cells already cultured in 100μl/ well (1:10 final dilution) for 1 hr. The relative number of cells was determined by measuring the absorbance at 450 nm.

Clonogenic formation assay

Cells (1×10^4 cells/well) in 12-well plates were grown in the presence of different concentrations of glucose or the indicated inhibitors for 14 days. The colonies were fixed and stained with 30% ethanol containing 1% crystal violet for 30 mins, and then were washed with ddH_2O.

Autophagosome formation assay

Cells seeded in 6-well plates were cultured with different glucose concentrations for 24 hr and were then stained with Cyto-ID® autophagy green dye (Enzo Lifesciences, Farmingdale, NY, USA) at 37 °C for 1 hr. Cells were washed with PBS three times and then fixed with 4% formaldehyde at room temperature for 20 min. The signal of autophagosome was detected by ECHO Revolve (San Diego, CA, USA) or measured

in BD FACSCalibur.

Cell cycle analysis

Cell seeded in 6-well plates were cultured with different glucose concentrations or treated with various inhibitors for 24 hr and fixed with ice-cold 70 % ethanol overnight at –20 °C. The cells were spun down and washed with PBS twice, then were stained with propidium iodide (PI) solution (1ml mix of 200 μg/ml RNAse and 50 μg/ml PI in PBS) at 37 °C for 30 min with the protection from light. The subpopulation of subG1 was measured in BD FACSCalibur (BD Biosciences, San Jose, CA, USA).

Immunoprecipitation (IP) and Western blot (WB) analysis

Total lysates were prepared with lysis buffer (4 M NaCl,1 M Tris, pH8.0), 10% SDS, Triton X-100, 10% sodium deoxycholate, 0.5 M EDTA), briefly sonicated, and then centrifugated at 15,000 rpm for 20 min at 4 °C followed by the collection of supernatants. For immunoprecipitation, one mg of total lysate incubated with primary antibody for overnight followed the incubation with protein A/G beads for 4 hours at 4 °C. The immunoprecipitates were washed with IP buffer (1 M HEPES, 1 M KCl, 1 M $MgCl_2$, 5 M NaCl) and eluted with sample dye. Total lysate or immunoprecipitates were subjected to protein separation in 8% or 12% of SDS-PAGE followed by protein transfer to polyvinyl difluoride (PVDF) or nitrocellulose (NC) membranes. The membranes were blocked with 5% milk for 1 hr at room temperature and incubated with primary antibodies at 4 °C overnight followed by the incubation with secondary antibodies in 5% milk for 1 hr at room temperature. The protein amount was developed with enhanced chemiluminescence (Bio-Rad Laboratories, Hercules, CA,

USA) reagent and detected in a chemiluminescence system.

Measurement of extracellular acidification rate (ECAR)

The ECAR in lung cancer cells and their ER clones were assessed by using a Seahorse XF®24 Analyzer (Agilent Technologies Inc., Santa Clara, CA, USA). Assays were performed according to the manufacturer's instructions. In brief, cells (2.5×10^4 cells/well) in 24-well XF microplate (Seahorse Biosciences, VIC, Australia) were cultured in glucose-free seahorse XF assay medium. Specific inhibitors, different glucose concentrations, and uncouplers were prepared in XF assay media following the experiment's design for sequential addition at the appropriate final concentrations (10 mM glucose, 1μM oligomycin, and 50 mM 2-DG). The data were normalized with cell numbers.

2-[N-(7-Nitrobenz-2-oxa-1,3-diazol-4-yl) amino]-2-deoxy-D-glucose (2-NBDG) uptake assay

Cells seeded in 6 well plates were pretreated with the indicated inhibitors for 3 days followed by the incubation with glucose-free media for 4 hr and 2-NBDG (100 μM/mL; Cayman, Ann Arbor, MI, USA) in PBS for 20 min at 37 °C. The uptake of 2-NBDG was detected by ECHO Revolve or measured in BD FACSCalibur.

[14C]-α-methyl-D-glucopyranoside (αMDG) uptake assay

The active glucose uptake ability of cells was determined by measuring the uptake of α-MDG (PerkinElmer, MA, USA), which is a specific subtract for SGLT. Cells seeded in a 12-well plate were cultured with different glucose concentrations or infected with SGLT1 shRNA. After washed with PBS once, the cells were incubated

with Krebs–Ringer–Henseleit (KRH; 120 mM NaCl, 4.7 mM KCl, 1.2 mM $MgCl_2$, 2.2 mM $CaCl_2$, and 10 mM HEPES, pH 7.4 [with Tris]) solution containing [^{14}C]-αMDG (0.1 μCi/ml) for 40 min. Following wash with PBS three times, cells were lysed by 1% Triton and added 2 ml scintillation solution. Then the uptake of [^{14}C]-αMDG was counted and presented as counts per minute (CPM) value in Beckman LS6000 Scintillation Counter (GMI, Ramsey, MN, USA), and the data were normalized with the protein amounts.

Human NSCLC clinical specimens

The acquisition of tumor specimens from NSCLC patients treated with EGFR TKIs were approved by the Ethics Review Board of China Medical University Hospital (DMR101-IRB1-120). Informed written consent was obtained from patients. The tissues were fixed in 10% formalin and embedded in paraffin, and 5μm tissue slides were prepared for IHC staining.

Immunohistochemistry Staining

Five-micrometer thick paraffin wax mouse-tissue sections were dewaxing by xylene and rehydrated by different concentrations of ethanol. These mouse-tissue sections were incubated with the indicated antibodies overnight and then stained with polymer HRP-conjugated secondary antibodies for 30 min followed by reaction with diaminobenzidine (DAB; Leica, Wetzlar, Germany) for 30 sec or 1 min. These slides were counterstained with hematoxylin. According to the H-score system, the immune-intensity of tumor tissue was scored by calculating the percentage of positive cells at different staining intensity levels, and the final score is ranked from 0 to 300. The score of SGLT1 level over than 200 was defined as high expression.

Xenograft tumor growth assay

Animal experiments were performed following a protocol approved by the Institutional Animal Care and Use Committee of China Medical University and Hospital (No. 102-40-N). H292 cells (1×10^6 cells/mouse) were subcutaneously injected into the female severe combined immunodeficient (SCID) mice at 4 weeks of age, and the tumor size was measured with calipers once per week. Once the tumor size reached 100~200 mm^3, mice were treated orally with saline, erlotinib (50 mg/kg), phlorizin (20 mg/kg), LX4211 (60 mg/kg), or the indicated combination for 30 days. A549/Luc cells were intravenously injected into the SCID mice. Tumor volume, as indicated by luciferase intensity, was measured by the Lumina LT In Vivo Imaging System (IVIS; PerkinElmer Inc., Waltham, MA, USA).

Site-directed mutagenesis

The human EGFR T678A, EGFR S1046/47AA mutants were generated by using the QuikChange Site-Directed Mutagenesis kit (Agilent Technologies Inc., Santa Clara, CA, USA) following the manufacturer's protocol. The primers were listed in the Supplementary information. Each mutation was verified by DNA sequencing.

Transient Transfection

Cells with 80% of confluence were subjected to transfection by incubation with DNA/TransIT®-X-2 (Mirus Bio, Madison, WI, USA) complex (ratio of 1:1.2) in serum-free medium for 6 hours followed by the refreshment with complete medium. The cells were harvested and subjected to the experiments after 72 hours of transfection.

Gene silence with shRNA

The shRNA clones against the indicated human genes were purchased from the National RNAi Core Facility at Academia Sinica (Taipei, Taiwan). Briefly, cells were infected with the indicated viral shRNA at the multiplicity of infection (MOI) of 125 for 3 days. Cells were refreshed with complete medium and then further subjected to the indicated experiments.

Statistical analysis

Analyses of patient survival and progression-free rates were performed using GraphPad Prism 8. Other statistical analysis was performed by Sigma plot. Data are displayed as the means ± SEM. The significance of the difference between the experimental and control groups was assessed by Student's *t*-test. The difference was considered to be significant if the *P*-value was < 0.05.

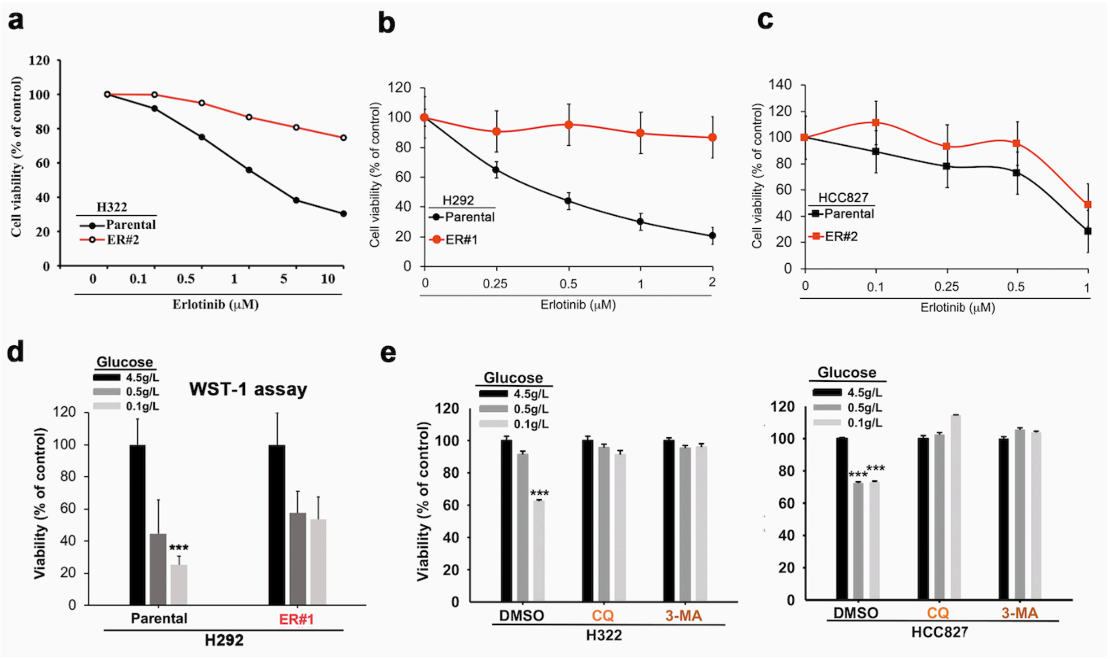

Supplementary Fig S1. The acquired erlotinib-resistant NSCLC cells were more tolerant to glucose deprivation. a-c. H322, H292, and HCC827 cells and their ER clones were treated with different concentrations of erlotinib. The viability was determined by MTT assay. **d**. H292 cells and their ER clone were cultured in different concentrations of glucose. The cell viability was measured in MTT. **e.** The effects of 3-MA or CQ on the glucose deprivation-induced cell death were examined in WST-1 analysis. Data in **(a-e)** represent as mean±s.d. from three independent experiments. **P* < 0.05; ****P*< 0.001 vs control group, Student's t test.

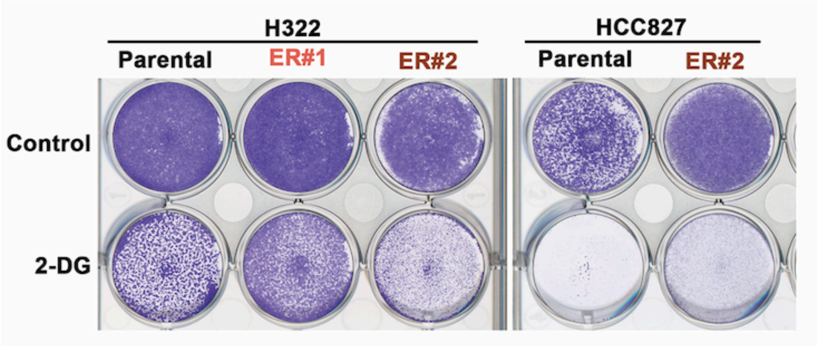

Supplementary Fig S2. Block of glycolysis decreased cell growth.

The inhibitory effects of 2-DG on the viability in H322 and HCC827 cells and their ER clones were measured by clonogenic assay.

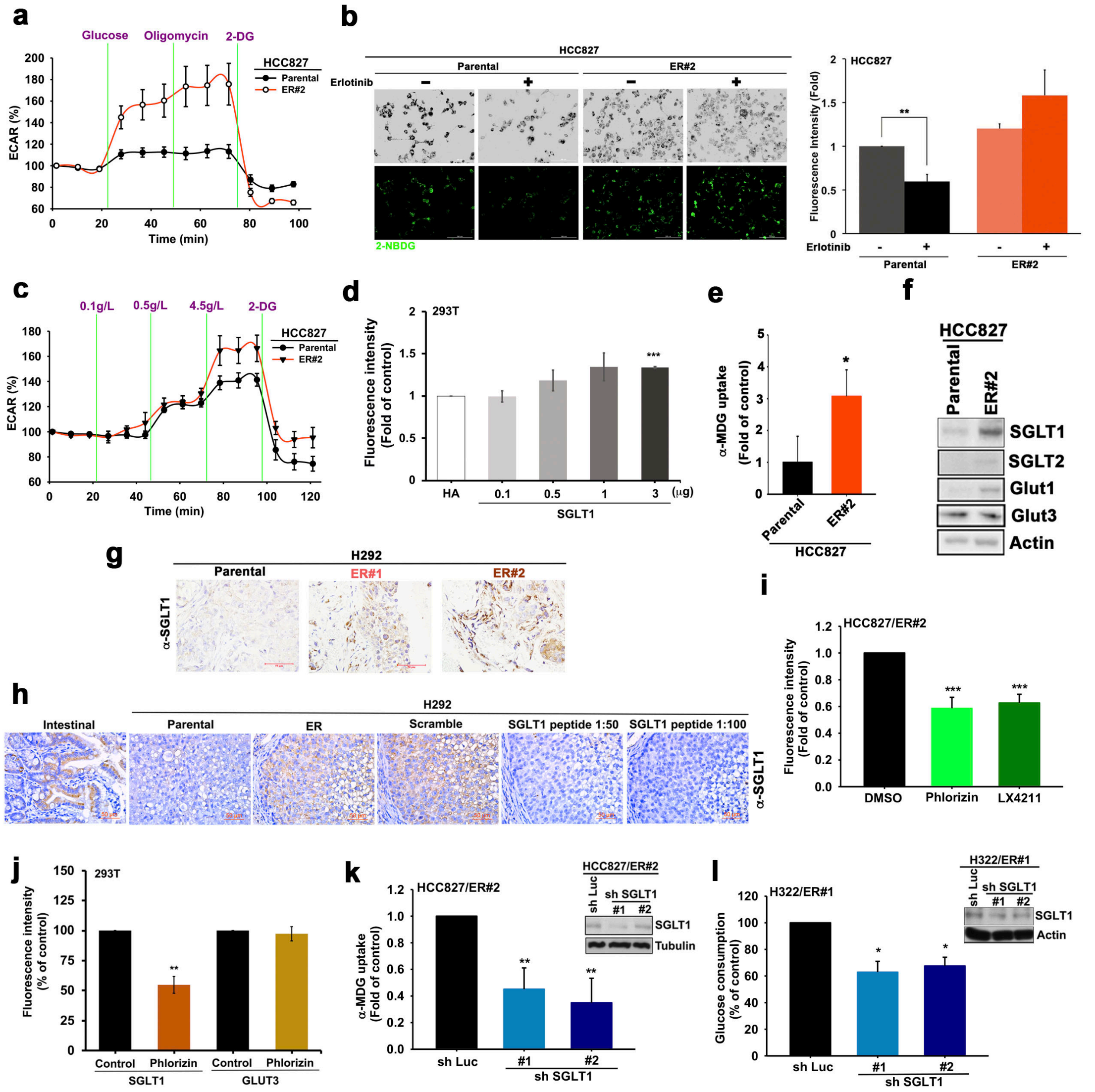

Supplementary Fig S3. The upregulated SGLT1 mediated glucose uptake of the acquired erlotinib-resistant cells. a. Changes in ECAR of HCC827 and their ER clones were measured by using the XF-24 Seahorse extracellular flux analyzer. **b.** 2-NBDG uptake ability of HCC827 and its ER clones under EGFR-TKI treatment were detected by immunofluorescent. Scale bar, 200 μm. **c.** Changes in ECAR in HCC827 and its ER clones in response to different glucose concentrations treatment were analyzed in XF-24 Seahorse extracellular flux analyzer. **d.** HEK-293T cells transfected with increasing amounts of SGLT1 cDNA were subjected to 2-NBDG uptake analysis. **e.** α-MDG uptake ability of HCC827 cells and their ER clones was detected by FACS and Beckman LS6000 Scintillation Counter. **f.** Protein levels of the

indicated glucose transporters in HCC827 and its ER clone cells were detected in WB with the indicted antibodies. **g and h**. Representative images of IHC staining of SGLT1 expression in the tumor sections from H292 and H292/ER cells (g) by using the specific anti-SGLT1 antibody which was validated with competitive peptide corresponding to the epitope sequence of SGLT1 (a.a.601-630) (h). Scale bar, 50 μm. **i.** The effects of 100μM phlorizin or 1μM LX4211 on 2-NBDG uptake ability in HCC827/ER#2 clones under a low glucose concentration condition were examined. **j.** The effects of phlorizin on 2-NBDG uptake ability of SGLT1- or Glut3-transfected HEK-293T cells under a low glucose concentration condition were examined. **k.** The effects of SGLT1 shRNA on the α-MDG uptake ability of HCC827/ER#2 clone were measured under low glucose condition by using Beckman LS6000 Scintillation Counter. **l**. The effects of SGLT1 shRNA on glucose consumption level of H322/ER#1 clone were analyzed. Data shown in **(a)**, and **(c-e),** and **(i-l)** represent as mean±s.d. from three independent experiments. *$P < 0.05$; **<0.01; ***$P < 0.001$ vs control group, Student's t test. Data in **(b)**, **(g)** and **(h)** are representative of three experiments.

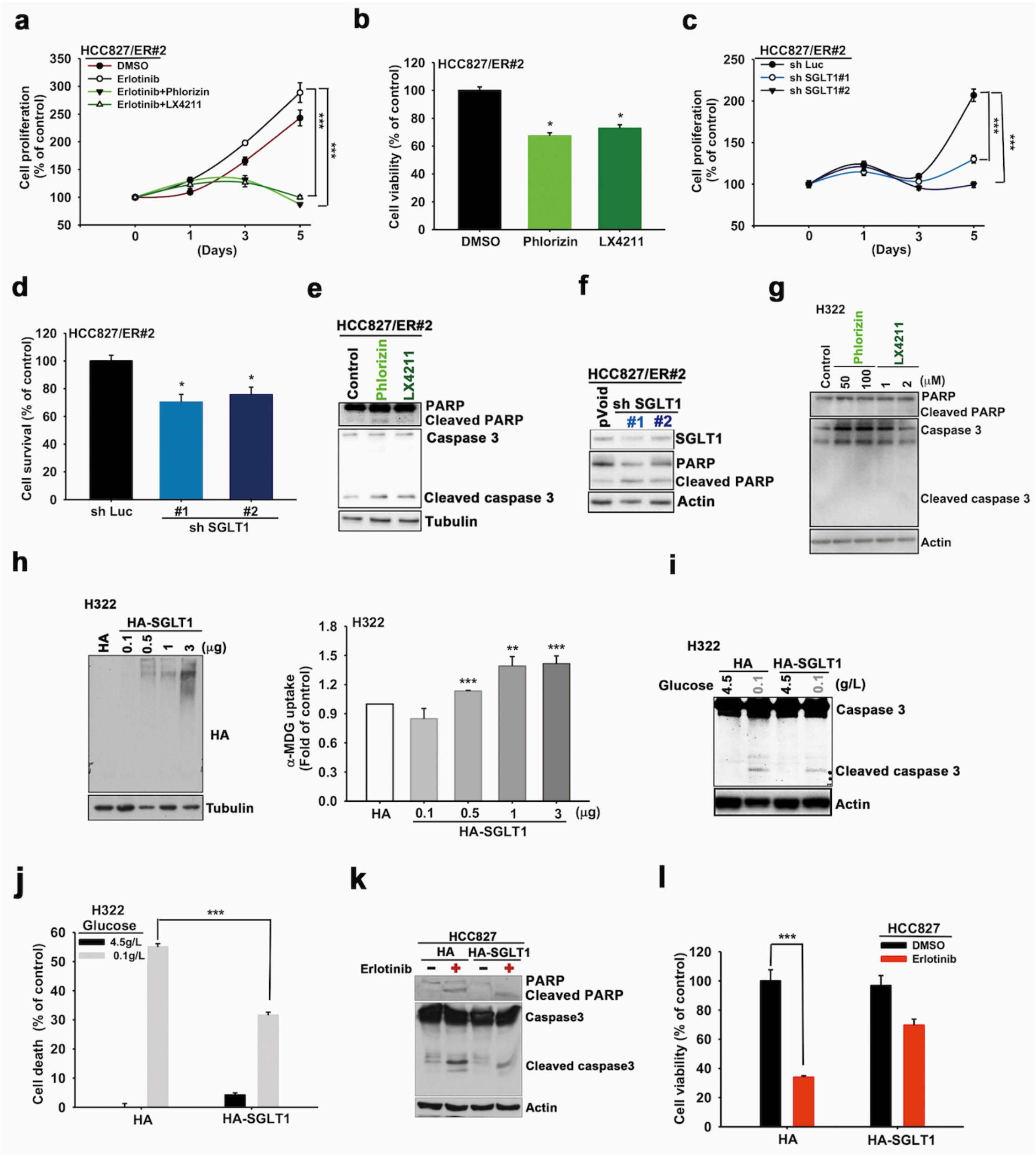

Supplementary Fig S4. The upregulated SGLT1 supported the cell viability of the acquired TKI-resistant cells. a. The cell proliferation of HCC827/ER clones in response to erlotinib, phlorizin, or LX4211 were determined in WST-1 analysis. **b.** The effects of phlorizin or LX4211 on cell viability of HCC827/ER#2 clones under low glucose concentration were measured in WST-1 analysis. **c and d.** The effects of SGLT1 shRNA on cell proliferation (c) and viability (d) of HCC827/ER#2 clones under low glucose concentration were determined in cell counting and WST-1 analyses, respectively. **e and f.** The effects of SGLT1 inhibitors (e) and shRNA (f) on

PARP cleavages, caspase 3 in HCC827/ER#2 clones were analyzed by WB. **g.** The effects of SGLT1 inhibitors on PARP cleavages, caspase 3 in H322 cells were analyzed by WB. **h**. H322 cells transfected with different amounts of SGLT1 cDNA were subjected to analyze the SGLT1 protein level in WB (left) and to measure α-MDG uptake (right). **i**. The effects of SGLT1 overexpression on caspase 3 cleavage induced by glucose deprivation in H322 cells were analyzed by WB. **j**. The relative cell death of SGLT1-expressing H322 cells in response to glucose deprivation was measured in WST-1 analysis. **k and l.** The effects of SGLT1 overexpression on the erlotinib-induced PARP and caspase 3 cleavages (k) and cell death (l) in HCC827 cells were analyzed in WB and WST-1 analyses, respectively. Data in **(a-d)**, **(h), and (j)** represent the mean and s.d. from three independent experiments. * $p< 0.05$; ***$p< 0.001$ vs control group, Student's t test. Data in **(e-g), (i) and (k)** are representative of three experiments.

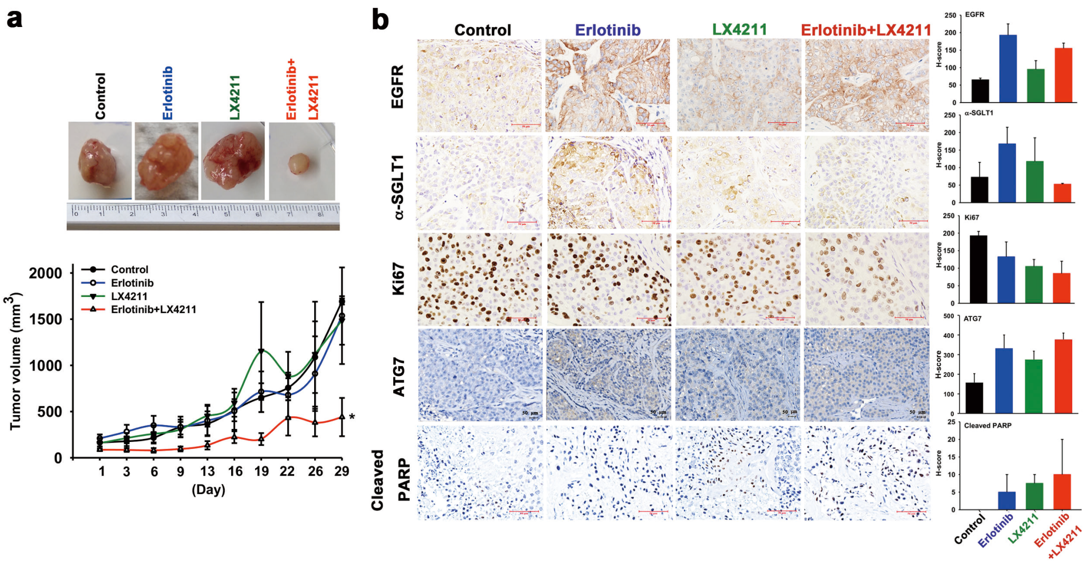

Supplementary Fig S5. Targeting SGLT1 reduced the development of acquired resistance to EGFR TKI *in vivo*. a. The growth rate of xenograft tumors of H292 cells in response to treatments with erlotinib, LX4211, or the combination of erlotinib and LX4211 was determined by measuring the tumor size. **b**. Representative IHC staining of xenograft tumors from (a) was performed with the indicated antibodies, and the H-score of protein expression was shown. Scale bar, 50 μm. Data represent mean±s.d. * $p < 0.05$ vs control group, Student's t test.

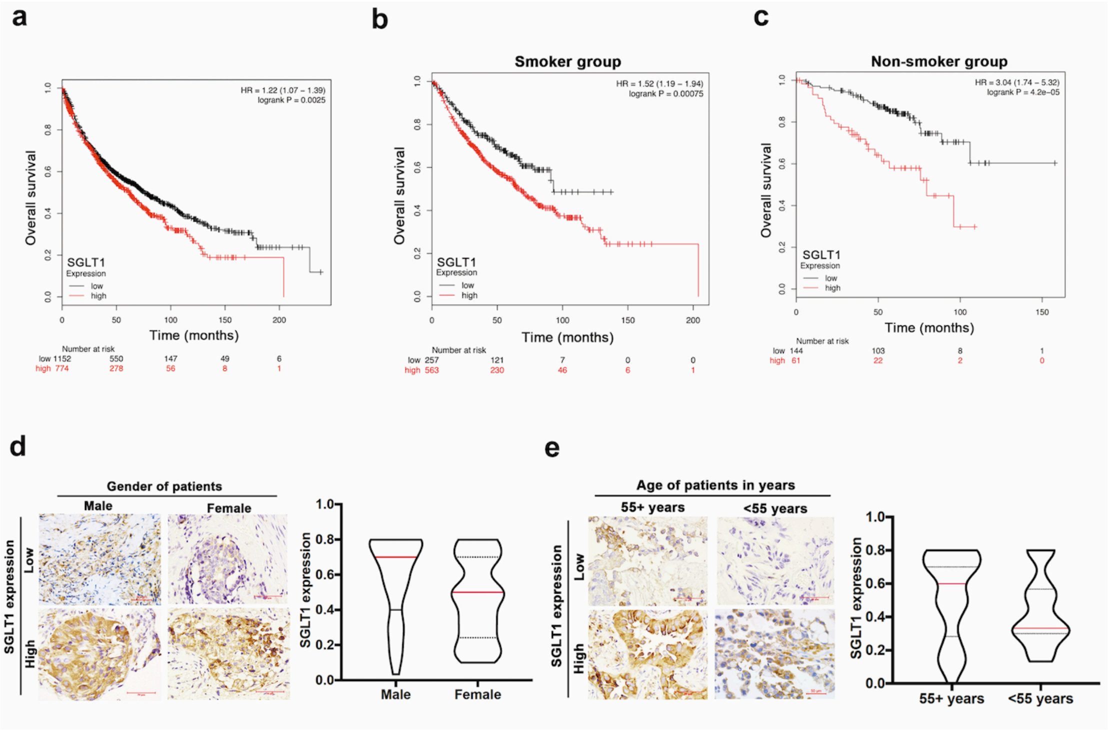

Supplementary Fig S6. SGLT1 expression negatively correlates with the clinical benefits of EGFR TKI in NSCLC patients. **a.** The clinical correlation of SGLT1 mRNA expression with overall survival rate was analyzed in the Kaplan Meier analysis. **b, c.** The SGLT1 mRNA expression was further classified into with (b) and without cigarette smoke (c) groups for Kaplan-Meier overall survival. **d, e.** The SGLT1 protein level in the paired tissues from treatment-naïve tumors and acquired TKI-resistant tumors of 9 lung cancer patients were examined by IHC staining and quantitated. The representative data was shown according to the gender (d) and age (e). Scale bar, 50 μm.

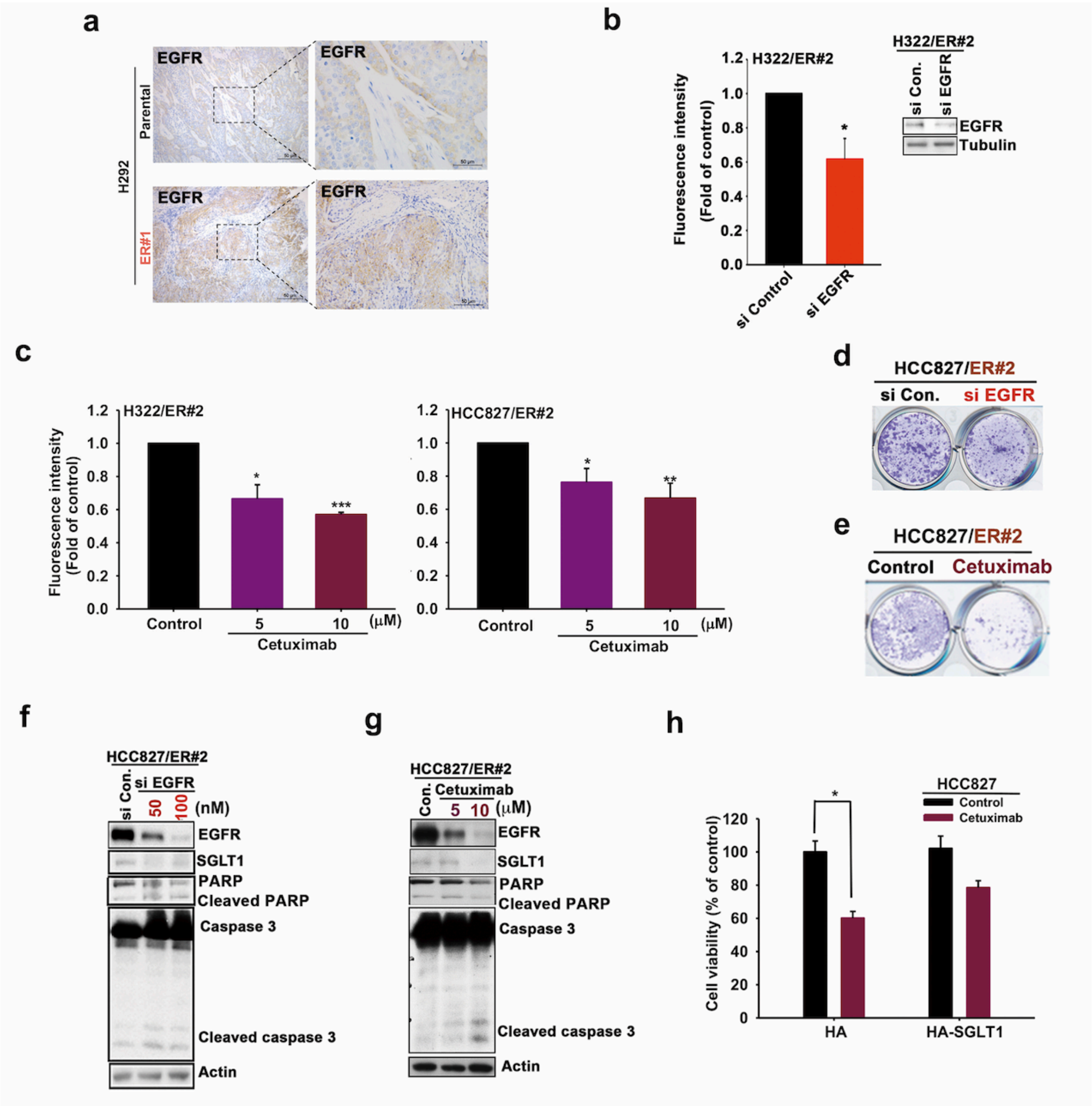

Supplementary Fig S7. **The increased EGFR mediated the glucose uptake and viability of the acquired erlotinib-resistant cells through SGLT1 upregulation. a**. The EGFR protein staining of H292 cells-xenograft tumor sections in response to erlotinib treatment was performed in IHC analysis. Scale bar, 50μm. **b-g**. The effects of monoclonal antibody cetuximab or EGFR siRNA on 2-NBDG uptake (b and c), colony formation (d and e), and caspase and PARP cleavages (f and g) of HCC827/ER#2 cells were determined, respectively. **h**. The effects of SGLT1 overexpression on the cetuximab-induced viability inhibition of HCC827 cells were examined in WST-1 analysis. Data in **(b and c)**, and **(h)** represent as mean±s.d. from three independent experiments. * $p < 0.05$; ** $p < 0.01$; *** $p < 0.001$ vs control group,

Student's t test. Data in **(a)** and **(d-g)** were representative of three experiments.

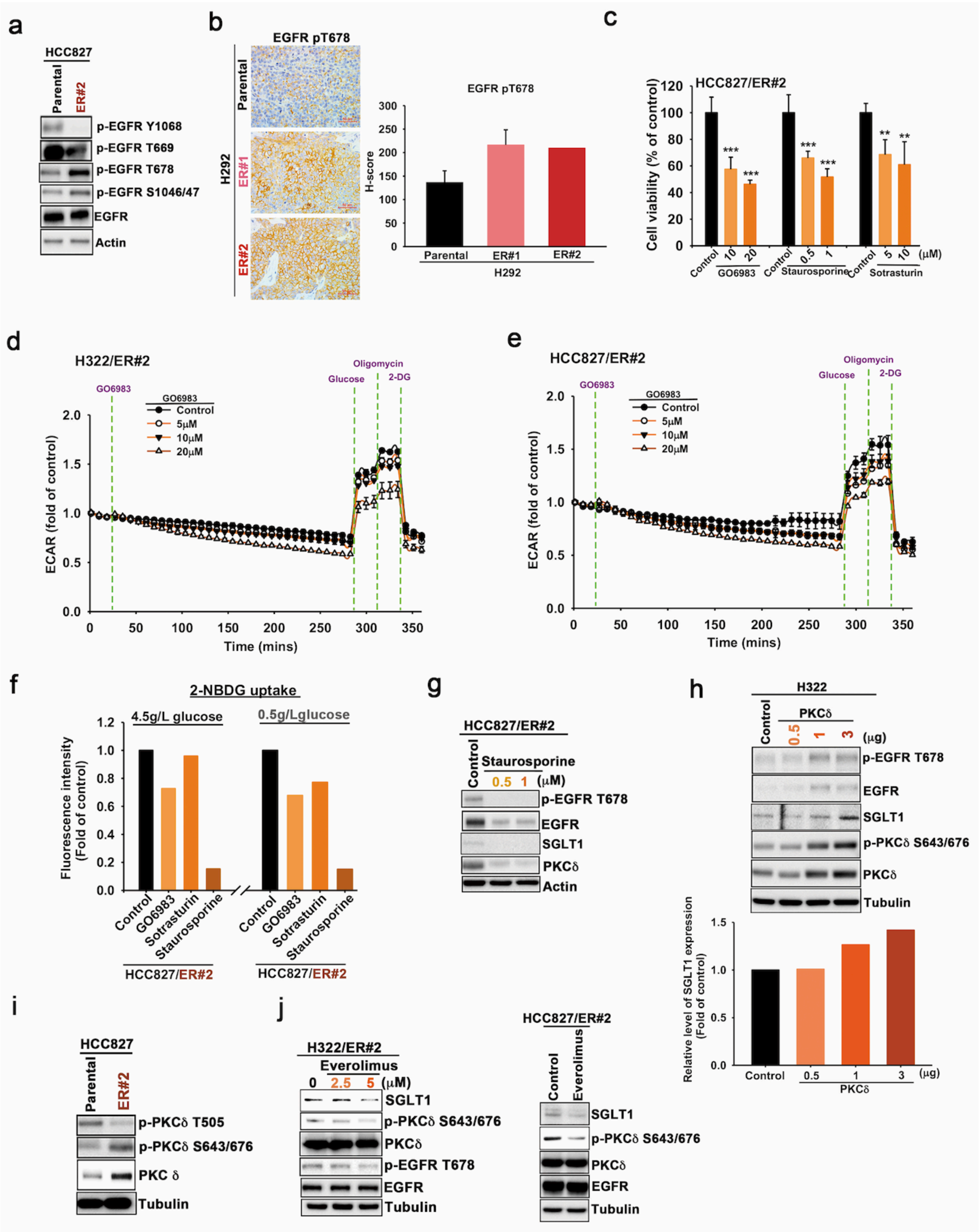

Supplementary Fig S8. **EGFR Thr678 phosphorylation by PKC delta mediated the SGLT1/EGFR interaction for SGLT1 protein stabilization. a**. The protein and phosphorylations of EGFR in HCC827 cells and their ER clones were analyzed in WB analysis. **b**. Representative IHC image and H-score of EGFR p-T678 expression in xenograft tumor sections of H292 cells and their ER clones were shown. Scale bar, 50 μm. **c**. The effects of GO6983, staurosporine, sotrasturin or HBDDE on cell

viability were determined in WST-1 assay. **d, e.** Changes in ECAR in H322/ER#2 (d) and HCC827/ER#2 (e) cells in response to GO6983 treatment for 4 hrs were analyzed in XF-24 Seahorse extracellular flux analyzer. **f**. The effects of various PKC inhibitors treatment on 2-NBDG uptake ability were analyzed by FACS analysis. **g-j.** The total lysates from staurosporine-treated HCC827/ER#2 cells (g), PKCδ-transfected H322 cells (h), HCC827 cells and their ER clones (i), and everolimus-treated H322/ER#2 cells (j) were subjected to WB analysis with the indicated antibodies. Data shown in **(c)**, **(d)**, and **(e-f)** represent as mean±s.d. from three independent experiments. ***$p < 0.001$ as compared with control group using Student's t test. Data in **(a)**, **(b)**, and **(g-j)** were representative of three experiments.

Table 1. Association of SGLT1 with clinical characteristics

		Analysis patients' number	**SGLT1**		*p* value
		Total number (72)	High	Low	
Gender					
	Male	35	18 (51.4%)	17 (48.5)	
	Female	37	9 (24.3%)	28 (75.6)	0.018*
Age (years)					
	≧55	53	26 (49.0%)	27 (50.9%)	
	<55	19	2 (10.5%)	17 (89.4%)	0.003**
Smoke					
	Smoker	26	12 (46.1%)	14 (53.8%)	
	Non-smoker	46	15 (32.6%)	31 (67.3%)	0.254
EGFR status					
WT		33	9 (27.2%)	24 (72.7%)	
Mutation		39	18 (46.1%)	21 (53.8%)	0.099
	del.19	20	10 (50%)	10 (50%)	
	L858R	16	7 (43.7%)	9 (56.2%)	
	del.19/T790M	1	0 (0%)	1 (100%)	
	L816Q	1	1 (100%)	0 (0%)	
	exon 20	1	0 (0%)	1 (100%)	0.552
Clinical T-stage					
	Tx-T0	5	2 (40%)	3 (60%)	
	T1-T2	14	7 (50%)	7 (50%)	
	T3-T4	44	16 (36.3%)	28 (63.6%)	0.662
Clinical N-stage					
	x	4	2 (50%)	2 (50%)	
	0	11	6 (54.5%)	5 (45.4%)	
	1	3	0 (0%)	3 (100%)	
	2	20	9 (45%)	11 (55%)	
	3	26	8 (30.7%)	18 (69.2%)	0.37
Pathological staging (AJCC)					
	Stage I	0	0 (0%)	0 (0%)	
	Stage II	1	1 (100%)	0 (0%)	
	Stage III	5	0 (0%)	5 (100%)	
	Stage IV	44	19 (43.1%)	25 (56.8%)	0.081
TKI drug therapy					
	Gefitinib	36	16 (44.4%)	20 (55.5%)	
	Erlotinib	35	13 (37.1%)	22 (62.8%)	

	Afatinib	2	0 (0%)	2 (100%)	0.417
TKI response CT					
	PR	20	8 (40%)	12 (60%)	
	SD	9	3 (33.3%)	6 (66.6%)	
	PD	38	16 (42.1%)	22 (57.8%)	0.89

*$P < 0.05$ and *$P < 0.01$
